# Combining Web-Based Attentional Bias Modification and Approach Bias Modification as a Self-Help Smoking Intervention for Adult Smokers Seeking Online Help: Double-Blind Randomized Controlled Trial

**DOI:** 10.2196/16342

**Published:** 2020-05-08

**Authors:** Si Wen, Helle Larsen, Marilisa Boffo, Raoul P P P Grasman, Thomas Pronk, Joeri B G van Wijngaarden, Reinout W Wiers

**Affiliations:** 1 Department of Psychology University of Amsterdam Amsterdam Netherlands; 2 Department of Psychology Education and Child Studies Erasmus University Rotterdam Rotterdam Netherlands; 3 Max Planck Institute for Brain Research Frankfurt Germany

**Keywords:** cognitive bias modification, attentional bias modification, approach bias modification, Web-based intervention, smoking addiction, smoking cessation, eHealth, randomized controlled trial

## Abstract

**Background:**

Automatically activated cognitive motivational processes such as the tendency to attend to or approach smoking-related stimuli (ie, attentional and approach bias) have been related to smoking behaviors. Therefore, these cognitive biases are thought to play a role in maintaining smoking behaviors. Cognitive biases can be modified with cognitive bias modification (CBM), which holds promise as an easy-access and low-cost online intervention. However, little is known about the effectiveness of online interventions combining two varieties of CBM. Targeting multiple cognitive biases may improve treatment outcomes because these biases have been shown to be relatively independent.

**Objective:**

This study aimed to test the individual and combined effects of two web-based CBM varieties—attentional bias modification (AtBM) and approach bias modification (ApBM)—in a double-blind randomized controlled trial (RCT) with a 2 (AtBM: active versus sham) × 2 (ApBM: active versus sham) factorial design.

**Methods:**

A total of 504 adult smokers seeking online help to quit smoking were randomly assigned to 1 of 4 experimental conditions to receive 11 fully automated CBM training sessions. To increase participants’ intrinsic motivation to change their smoking behaviors, all participants first received brief, automated, tailored feedback. The primary outcome was point prevalence abstinence during the study period. Secondary outcomes included daily cigarette use and attentional and approach bias. All outcomes were repeatedly self-assessed online from baseline to the 3-month follow-up. For the examination of training effects on outcome changes, an intention-to-treat analysis with a multilevel modeling (MLM) approach was adopted.

**Results:**

Only 10.7% (54/504) of the participants completed all 11 training sessions, and 8.3% (42/504) of the participants reached the 3-month follow-up assessment. MLM showed that over time, neither AtBM or ApBM nor a combination of both differed from their respective sham training in point prevalence abstinence rates (*P*=.17, *P*=.56, and *P*=.14, respectively), and in changes in daily cigarette use (*P*=.26, *P*=.08, and *P*=.13, respectively), attentional bias (*P*=.07, *P*=.81, and *P*=.15, respectively), and approach bias (*P*=.57, *P*=.22, and *P*=.40, respectively), while daily cigarette use decreased over time across conditions for all participants (*P*<.001).

**Conclusions:**

This RCT provides no support for the effectiveness of combining AtBM and ApBM in a self-help web-based smoking cessation intervention. However, this study had a very high dropout rate and a very low frequency of training usage, indicating an overall low acceptability of the intervention, which precludes any definite conclusion on its efficacy. We discuss how this study can inform future designs and settings of online CBM interventions.

**Trial Registration:**

Netherlands Trial Register NTR4678; https://www.trialregister.nl/trial/4678

## Introduction

### Background

Smoking is one of the major risk factors for preventable diseases and premature deaths [1]. Although most smokers are aware of the health risks of smoking and desire to quit, almost 80% of those who attempt to quit relapse within 3 months [2]. An important factor in addictive behaviors concerns automatically activated cognitive motivational processes, which are difficult to inhibit via reflective processes aimed at long-term health outcomes [3-5]. As a result, addictive behaviors might interact with substance-related cue-driven reactions, such as relatively automatic cognitive biases.

Smokers have been found to selectively pay more attention to smoking-related cues in the environment (ie, smoking-related attentional bias) and to impulsively reach out to these smoking-related cues (ie, smoking-related approach bias [6-9]). These biases have been related to the urge to smoke, the severity of nicotine dependence, and relapse rates [8-11]. Therefore, smoking-related cognitive biases are thought to be one of the mechanisms underlying smoking behaviors, highlighting the importance of targeting them in smoking cessation interventions.

Varieties of cognitive bias modification (CBM) have been developed to directly target the cognitive biases [12], such as attentional bias modification (AtBM, usually delivered with a modified visual probe task, VPT [13]) and approach bias modification (ApBM, usually delivered with a modified approach-avoidance task, AAT [14]). In the addiction field, the clinical effects of CBM as a behavior change intervention (as opposed to proof-of-principle studies [15]) were first tested in the alcohol domain. Several pioneering randomized controlled trials (RCTs) in clinical samples showed that multiple sessions of AtBM [16] or ApBM [17,18] were more effective than the respective sham training in reducing the targeted alcohol-related cognitive bias and relapse rates, when provided as an add-on to the regular cognitive behavioral therapy (CBT). Furthermore, ApBM training had effects on the reduced relapse rates that were mediated by changes in alcohol-related approach bias [17,19]. Therefore, based on both theory and the available evidence, CBM has shown the potential to be an effective novel intervention in the alcohol addiction domain.

A key advantage of CBM interventions is that they are delivered as computerized tasks, which are easily administered online, featuring CBM as a potential easy-access and low-cost online intervention, particularly for the smoker population. Instead of attending formal smoking cessation programs [20], smokers often search for online help to quit smoking [21,22]. Therefore, we designed a web-based CBM intervention specifically for smokers seeking help online. In the pioneering studies referenced above, the interventions only targeted 1 cognitive bias. However, addictive behaviors are characterized by multiple relatively independent cognitive biases [23]. Thus, combining multiple CBM varieties that target different cognitive biases may enhance the treatment outcomes by combining their effects, as well as through potential synergistic effects. Note that, at the time we set up this study (ie, in 2013), there was only 1 protocol study combining different web-based CBM varieties in an intervention targeting alcohol use disorder [24], and no studies had yet investigated the effectiveness of a combined web-based CBM intervention for smokers.

Since 2013, some studies have explored the clinical effects of CBM as a behavior change intervention for smoking problems, although the evidence is still limited (see Mühlig et al [25] for a narrative review and Boffo et al [26] for a Bayesian meta-analysis). In total, 3 RCTs delivered CBM in an online setting and 5 in a clinical or laboratory setting. Regarding the CBM studies in online settings, 1 RCT showed that web-based ApBM alone could produce specific effects on reducing smoking in adult smokers [27], while 2 other RCTs did not support that multiple sessions of web-based ApBM alone [28] or web-based AtBM alone [11] were effective in promoting smoking cessation in adult smokers, although in the latter study, AtBM positively affected continued abstinence at the 6-month follow-up in a subgroup of heavy smokers [11]. Regarding the CBM studies in clinical or laboratory settings, 1 RCT showed that multiple sessions of ApBM plus CBT led to larger reductions in daily cigarette consumption in inpatient psychiatric smokers than sham training [29], while 4 other RCTs reported that multiple sessions of ApBM plus CBT [30,31], AtBM alone [32], or plus nicotine patches and behavioral support [33] did not result in better smoking treatment outcomes than sham training in smokers who intended to quit. In sum, evidence for the effectiveness of AtBM and ApBM in the smoking addiction domain is mixed. Therefore, more research is needed to investigate whether AtBM together with ApBM can benefit smoking cessation interventions.

### Study Design, Objectives, and Hypotheses

**Figure 1:**
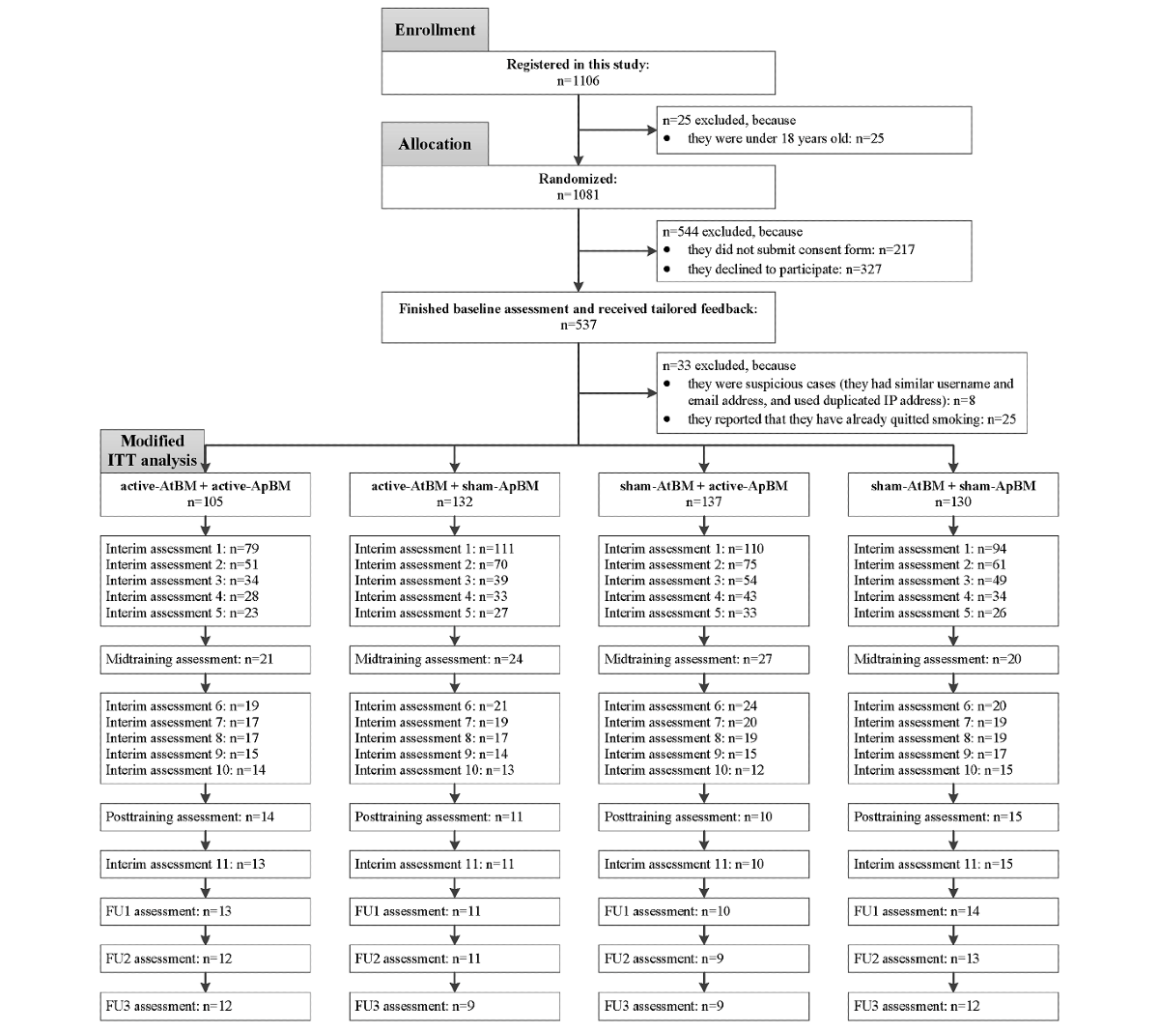
Consolidated Standards of Reporting Trials flow diagram. The number of participants reported here is based on timeline follow back data for our primary outcome. A similar dropout pattern is observed for all other outcomes. AtBM: attentional bias modification; ApBM: approach bias modification; Interim assessment: brief assessment before each training session; FU1, FU2, FU3: follow-up assessment at 1, 2, and 3 months; ITT: intention-to-treat; IP: internet protocol.

This study aimed at investigating the individual and combined effects of 2 varieties of web-based CBM, AtBM and ApBM, in adult smokers who were seeking online help for quitting smoking. A double-blind RCT was conducted with a 2 × 2 factorial design, in which participants received 11 fully automated sessions of either an active or a sham version of both types of CBM training, resulting in 4 experimental conditions (active-AtBM + active-ApBM; active-AtBM + sham-ApBM; sham-AtBM + active-ApBM; sham-AtBM + sham-ApBM; Figure 1). To increase participants’ intrinsic motivation to change their smoking behaviors before the CBM training, all participants first received brief, automated, tailored feedback, irrespective of their CBM condition. The primary outcome was point prevalence abstinence (PPA), while the secondary outcome included changes in daily cigarette use (DCU). Progressive changes in attentional bias and approach bias were also included as secondary cognitive outcomes to verify that the CBM trainings actually changed the targeted cognitive process. All outcomes were repeatedly assessed from baseline to the 3-month follow-up. We hypothesized that, compared with its respective sham training, each type of active CBM training would (1) be more effective in fostering PPA and in decreasing DCU and (2) lead to larger decreases in the specific cognitive bias it targeted. Given that AtBM and ApBM may tap into a separate process [23], we also hypothesized that (3) the condition with double active CBM trainings would be the most effective in changing smoking-related outcomes.

Since craving, depression severity, and motivation to quit smoking have been found to be related to cognitive biases or smoking behaviors [34-36], these variables were also included in this study as additional secondary outcomes. Furthermore, we explored whether participants were aware of which version of each CBM training they received (ie, the active or the sham version) and whether this moderated training effects. The methods and results for the additional secondary outcomes and the exploratory moderation analyses are reported in Multimedia Appendices 1 and 2.

## Methods

### Participants and Procedure

#### Participant Enrollment

Adult smokers were recruited across the Netherlands through our lab website (Addiction Development and Psychopathology Lab of the University of Amsterdam, ADAPT [37]), press releases (eg, TV interviews, newspapers, and scientific books [38]), and word-of-mouth communication. The ADAPT website is open-access and provides a series of cognitive training targeting a variety of addiction and affective-related problems such as smoking, alcohol, gambling, anxiety, and depression. We started to recruit participants from June 2013. Since this was the first study to test the effectiveness of 2 combined web-based CBM trainings as a behavior change intervention for smoking cessation, no effective knowledge (eg, CBM training effect sizes and dropout rates) was available for us to calculate the sample size at the time we set up the study. Therefore, our aim was to recruit as many participants as possible with the minimum of at least 75 participants per training condition. We stopped the recruitment in December 2018.

Interested participants were directed to the study website [39]. The website explained the scientific rationale of CBM training, training to overcome mental habits and automatic responses to smoking, and explained that the current intervention program combined 2 types of CBM freely available for people who would like to quit smoking. As the study concerned a self-help intervention open to everybody, there were no specific inclusion criteria, except for being aged 18 years and older and able to understand Dutch (the intervention was only provided in Dutch).

Upon registration, participants created their own user account by providing their username, password, and email address, were screened regarding their age, and were then randomized (see the Randomization and Blinding section). Participants were notified of their eligibility via email. Eligible participants who clicked on their emailed link were returned to the study website where they submitted a consent form. Through the consent form, participants were fully informed about the whole study procedure, that the effectiveness of the 2 CBM trainings was being tested (compared with 2 training types where no or smaller effects were expected, ie, sham training), and that they had a 25% chance to be assigned to the condition with 2 sham trainings. Afterward, participants completed the baseline assessment, at the beginning of which they received brief, automated, tailored feedback.

Since the enrollment occurred online, additional actions were taken to check multiple identities. Participants who used similar usernames, email addresses, and internet protocol addresses were excluded in the data analysis stage (n=8). In addition, participants who self-reported that they already quit smoking before the training were also excluded in the data analysis stage (n=25; Figure 1).

#### Training Procedure

After the baseline assessment, participants were invited to complete 11 CBM training sessions and assessments at midtraining, posttraining, and follow-ups at 1, 2, and 3 months, respectively. The midtraining assessment took place between training sessions 5 and 6; and the posttraining assessment took place between sessions 10 and 11. The 11th training session was a *mask* session to minimize self-presentation biases during the posttraining assessment. All training sessions were web-based, and all assessment sessions were self-assessed via web-based questionnaires and computerized tasks. Each training or assessment session automatically opened 24 hours after the previous session was completed and stayed open for 30 days. When each session opened, an automated notification was sent to the participants. If participants did not complete the session, they received an automated reminder email after 3 days, 7 days, 11 days, and 3 weeks until the session closed (including the baseline session). They were not allowed to skip sessions, were excluded from the study if they missed any of the sessions, and were advised to complete the 11 training sessions within 4 weeks (eg, 3 sessions per week). Participants were allowed to train on a daily basis and could arrange their own training schedule. They could contact the responsible researcher (the second author) by email in case they had questions or technical problems.

#### Debriefing and Compensation

After completing the 3-month follow-up assessment, participants were debriefed about their condition allocation via email. They were not compensated for participation. However, all participants had the opportunity to receive 11 booster training sessions if they completed the whole study procedure, consisting of the same 11 training sessions of double active CBM training without practice and mini-assessment blocks (see the Cognitive Bias Modification section).

#### Randomization and Blinding

The study was as a double-blind trial. Upon creation of a study account, participants were automatically randomized to 1 of the 4 training conditions by a computer randomization algorithm. The randomization was stratified by gender with a 1:1:1:1 ratio; therefore, participants were randomly assigned to one of the conditions to which the fewest participants of their gender had been allocated so far. Since the randomization was fully automated and conducted by a computer algorithm, allocation concealment was ensured. In addition, the automated study procedure ensured that participants were blinded to the training condition they were assigned to. The second author could access the database during the data collection to monitor the data collection process and was responsible to reply to participants in case they had questions or technical problems. The first author could access the database after data collection completion to download and analyze the data. No authors provided any treatment to the participants nor assessed any of the outcomes during the trial.

The study was approved by the Ethics Committee of Psychology at the University of Amsterdam (reference number: 2013-DP-3047) and registered in the Netherlands Trial Register (NTR4678).

### Intervention

#### Cognitive Bias Modification

In total, there were 11 CBM training sessions. Each training session consisted of 2 tasks used to both assess and retrain the cognitive biases, and each training session lasted about 20 to 25 min. Task settings of the assessment and the training version were the same, except for an additional built-in stimulus response contingency recasting the assessment task into training (see below). The order of task presentation was counterbalanced between participants and fixed within participants across all sessions. Task parameters (eg, stimulus onset, response time window, intertrial interval, etc) for both tasks were designed as in previous studies [24,40]. Two kinds of stimuli were used for both tasks: smoking-related (eg, somebody smoking or a package of cigarettes) and visually matched neutral pictures (eg, somebody holding pencils or a box of pencils; see Multimedia Appendix 3 for task stimuli).

#### Attentional Bias Assessment

To assess attentional bias (AtB), we used an online version of the VPT [13,24,40]. In each trial, participants had to respond to a probe (a small arrow pointing upward or downward) presented at the location of one of 2 stimuli (ie, a smoking-related and a neutral picture) displayed next to each other on the computer screen. In half of the trials, the probe appeared immediately after the 2 pictures disappeared (*after* format), measuring the early detection of smoking-related pictures (attention engagement). In the other half, the probe appeared on top of one of the 2 pictures, which stayed on screen (*top* format), measuring the relative difficulty to disengage from smoking-related pictures (attention disengagement) [40]. Participants were required to respond to the direction of the probe as fast as possible by pressing the corresponding keys on the keyboard (U and N). The probe direction was set randomly upward or downward with the restriction that up and down appeared equally often. To assess AtB, the probe followed smoking-related pictures (smoking trials) and neutral pictures (nonsmoking trials) equally often. It is assumed that discrimination of the probe direction will be quicker when probes appear in the locus that participants are already attending to, that is, in the case of smokers, on the smoking-related stimuli. The VPT included a practice block with 8 trials and an assessment block with 320 trials.

#### Attentional Bias Modification

To retrain AtB, we used a modified version of the VPT [24,40]. Participants in the active training condition were trained to shift their attention from smoking-related pictures to neutral pictures by exposing them only to nonsmoking trials (ie, the probe only followed the neutral pictures), whereas participants in the sham training condition were presented with 50% of smoking trials and 50% of nonsmoking trials (ie, continued assessment). Each AtBM session started with a practice block (8 trials) and a mini-assessment block (128 trials), after which, participants received the active or sham version of the AtBM (192 trials).

#### Approach Bias Assessment

To assess approach bias (ApB), we used an online version of the AAT [14,24,40]. In each trial, a smoking-related or a neutral picture rotated 3° to the right (right-format) or left (left-format) was presented in the middle of the computer screen. Participants were required to respond (pull or push away) to the format rather than the content of the picture as fast as possible by pressing the corresponding keys on the keyboard (U and N). The pull and push responses were accompanied by a zooming feature: pulled pictures enlarged in size and pushed pictures shrunk, generating the sense of approach and avoidance, respectively. The contingency between the picture format and the response (ie, rotation direction and pull or push response) was counterbalanced across participants. To assess ApB, smoking-related and neutral pictures were pushed and pulled equally often. It is assumed that (faster) approach rather than avoid responses would be triggered by appetitive or affective stimuli, that is, in the case of smokers, by the smoking-related stimuli. The AAT included a practice block with 12 trials and an assessment block with 160 trials.

#### Approach Bias Modification

To retrain ApB, we used a modified version of the AAT [24,40]. Participants in the active training condition were trained to avoid smoking-related pictures by exposing them only to smoking/push and neutral/pull trials, whereas participants in the sham training condition were presented with 50% pull and 50% push trials for both smoking-related and neutral pictures (ie, continues assessment). Each ApBM session started with a practice block (12 trials) and a mini-assessment block (64 trials), after which, participants received the active or sham version of the ApBM (192 trials).

### Automated Tailored Feedback

All participants received brief automated tailored feedback at the beginning of the baseline assessment. This session provided feedback based on participants’ current smoking behaviors, attitudes toward smoking, perceived importance, confidence, motivation to quit, and goals and plans to change smoking behaviors [41,42]. The tailored feedback consisted of (1) summarizing the information participants provided, (2) comparing their smoking behaviors and attitudes toward smoking with current smokers and ex-smokers, (3) challenging and modifying their positive attitudes toward smoking by providing health risk information, and (4) providing tips and support for their further changing progress.

### Assessment Measures

#### Outcomes

##### Primary Outcome

The primary outcome PPA was determined by using the timeline follow-back method (TLFB [43]) and was assessed at 6 main assessment time points (eg, baseline, midtraining, posttraining, and follow-ups at 1, 2, and 3 months) and 11 interim assessment time points (eg, a brief assessment before each training session). When TLFB was administrated at baseline and the follow-up assessments, participants reported the number of cigarettes they smoked per day in the past 7 days. When TLFB was administrated at midtraining, posttraining, and before each training session, participants reported the number of cigarettes they smoked per day since the last training or assessment session for a maximum of 7 days.

PPA was defined as not smoking at all over the period of reported days at each assessment time point and was coded as 1 (quit: the sum score of DCU=0) or 0 (not quit: the sum score of DCU >0). Note that the primary outcome preregistered was 7-day PPA at the follow-up assessments. That is, in the original plan, we focused on the medium-term training effects on the smoking status. However, because of the huge dropout rates (Figure 1), we decided to include all available data at all assessments. As a result, in the current report, we focused on the changes in the PPA over time.

##### Secondary Behavioral Outcome

The secondary behavioral outcome DCU was also derived from the TLFB data. DCU was calculated at each assessment time point by summing the number of cigarettes reported each day divided by the number of reported days. The internal consistency (Cronbach α) for the TLFB at baseline was .98.

##### Secondary Cognitive Outcomes

Secondary cognitive outcomes included AtB and ApB assessed with the online version of the VPT and AAT, respectively, described above (see the Cognitive Bias Modification section). Both biases were assessed at 4 main assessment time points (eg, baseline, midtraining, posttraining, and 3-month follow-up) and 11 interim assessment time points (eg, a mini-assessment block in each training session). By doing this, progressive changes in the cognitive biases over the study could be detected.

An AtB score for smoking was computed for both *after* and *top* trial formats by subtracting the median response time in smoking trials from that in nonsmoking trials. A positive score reflected an attentional bias toward smoking-related pictures, whereas a negative score reflected an attentional bias away from the smoking-related pictures and toward the neutral pictures.

An ApB score for each stimulus category was computed by subtracting the median response time in pull trials from that in push trials. A smoking-specific ApB score was defined as the difference between ApB scores for smoking-related pictures and neutral pictures. A positive score reflected an action tendency toward smoking-related pictures, whereas a negative score reflected an avoidance tendency for smoking-related pictures.

Bootstrapped split-half reliability estimates [44] for both VPT and AAT at baseline were obtained by using the *splithalf* package in R (version 0.3.1 [45]), which performed 5000 random splits. The reliability of VPT was *r*=0.25, 95% CI 0.19 to 0.31 (Spearman-Brown corrected *r*_sb_=0.40, 95% CI 0.32 to 0.48), and the reliability of AAT was *r*=0.02, 95% CI −0.10 to 0.13 (Spearman-Brown corrected *r*_sb_=0.03, 95% CI −0.18 to 0.23).

#### Other Measures

##### Baseline Measures

At baseline, demographics and smoking history information was collected, including age, gender, highest education level, marital status, household income/month, DCU in general, duration in terms of years of smoking, and previous quit attempts. Nicotine dependence was assessed with the Modified Fagerström Tolerance Questionnaire (mFTQ [46]). The internal consistency (Cronbach α) for the mFTQ was .71 in this study. Motivation to changing smoking behaviors was assessed with the Readiness to Change Questionnaire (RCQ [47,48]). The internal consistency (Cronbach α) for the RCQ was .64 in this study.

##### Training Evaluation and Reasons to Leave the Intervention

The training evaluation questions (TEQs) were administrated at the posttraining assessment, where participants evaluated both the CBM training as a whole and the AtBM and ApBM training. In addition, participants also indicated if they were aware of the training condition they were assigned to. For participants dropping out of the study before completing the posttraining assessment, TEQs could be triggered by the participants themselves when requiring to stop the study, by clicking on a web link included in the reminder emails they received. In this case, participants provided reasons for leaving the intervention, in addition to the training evaluation and their awareness of the training condition they were assigned to.

### Data Analysis

#### Task Data Preparation

Preparation of both VPT and AAT data can be found in Multimedia Appendix 4. For the VPT, there was no indication of a difference in AtB scores between *after* and *top* trials in the whole sample (see Multimedia Appendix 4 for details). Thus, we combined the 2 AtB scores (ie, engagement and disengagement AtB) into a single AtB index.

#### Preliminary Analyses

To check for baseline differences and differences in training compliance and retention across the 4 conditions, and to check for any differences in training evaluation between training dropouts and training completers, chi-square tests and one-way analysis of variance (ANOVA) were conducted. To verify if participants showed smoking-related AtB and ApB at baseline, one-sample *t* tests were conducted. To determine if AtB and ApB were correlated with smoking-related variables at baseline, zero-order Pearson correlations were computed.

#### Hypotheses Testing

To test the training effects, a multilevel modeling (MLM) approach was adopted, which allows for an intention-to-treat (ITT) analysis including all available data and takes the clustering of data by participants into account [49]. In our analyses, all models incorporated a random intercept for participants, used the maximum likelihood estimator, and were conducted in R with the *lme4* (version 1.1.17 [50]) and *lmerTest* packages (version 3.0.1 [51]). An alpha of .05 (two-sided) was applied to all hypotheses testing.

We used a piecewise step-function growth curve model [52] to track outcome changes over time. For PPA, the training effects were evaluated over 3 time phases: first half intervention phase (TP1: from baseline to midtraining assessment), second half intervention phase (TP2: from interim assessment 6 to posttraining assessment), and follow-up phase (TP3: from interim assessment 11 to the 3-month follow-up). Although all participants were coded as 0 (not quit) for PPA at baseline, modeling the growth curve starting at baseline is necessary for ITT analyses. Therefore, baseline was included into TP1. For DCU, AtB, and ApB, the training effects were evaluated over 4 time phases: baseline (TP1), first half intervention phase (TP2: from interim assessment 1 to midtraining assessment), and, similar to PPA, second intervention phase (TP3) and follow-up phase (TP4).

To test the training effects on PPA, a multilevel logistic regression analysis was conducted, whereas for DCU, AtB, and ApB, a series of multilevel linear regression analyses was conducted. Predictors included time phase, AtBM (active vs sham), ApBM (active vs sham), and their interactions. Our hypotheses testing focused on the two-way interaction effects of time phase × AtBM and time phase × ApBM, and the three-way interaction effects of time phase × AtBM × ApBM.

## Results

### Preliminary Analyses

#### Sample Description

The Consolidated Standards of Reporting Trials flow diagram is reported in Figure 1. The final sample comprised 504 adult smokers who were seeking online help for quitting smoking. Overall, the final sample had a mean age of 45.10 (SD 13.36) years. Of these, 66.9% (337/504) were female, 74.8% (377/504) were highly educated (bachelor’s degree or above), 72.8% (367/504) were unmarried, and 42.5% (214/504) had a monthly household income above the national modal income of about €3000 (US $3329). On average, participants had smoked for 27.55 (SD 13.60) years, used to smoke 17.01 cigarettes per day (SD 8.83), had a medium level of nicotine dependence (mFTQ: mean 3.21, SD 1.62, range 0-6). In addition, on average, participants had made 5.70 previous quit attempts (SD 5.22) and were highly motivated to quit before training (RCQ: mean 12.10, SD 5.41, range −24 to 24).

Baseline characteristics of the final sample per condition can be found in Table 1. Overall, participants’ baseline characteristics did not differ among the training conditions, with the exception of gender (Table 1). Adding gender as a covariate to the models did not affect the relative model fit or the significance of any relevant parameters.

#### Adherence and Retention

On average, participants completed 2.75 (SD 3.45) out of the 11 training sessions, which did not differ between conditions (*F*_3,500_=0.11; *P*=.95). Of the final sample, 67.1% (338/504) completed at least one training session, 21.4% (108/504) completed at least five training sessions, and 10.7% (54/504) completed all 11 training sessions, all of which did not differ between conditions (χ^2^_3_=2.5, *P*=.48; χ^2^_3_=0.9, *P*=.83;χ^2^_3_=1.5, *P*=.68). On average, the training interval was 5.57 (SD 4.64) days, which did not differ between conditions (*F*_3,332_=1.32; *P*=.27).

Regarding the evaluation of retention, 2 measures were considered (Figure 1). First, only 61.8% (529/856) of the eligible participants completed the baseline assessment, which did not differ between conditions (χ^2^_3_=7.4; *P*=.06). Second, for the final sample, only 18.3% (92/504), 9.9% (50/504), and 8.3% (42/504) of the participants completed the primary outcome measure (ie, TLFB) at midtraining, posttraining, and follow-ups, respectively. All retention rates did not differ between conditions (χ^2^_3_=1.1, *P*=.77; χ^2^_3_=3.2, *P*=.37; χ^2^_3_=2.4, *P*=.49).

#### Training Evaluation and Quitting the Intervention

In total, 19.6% (99/504) of the final sample provided the training evaluation. This subsample was older (*F*_1,502_=15.67; *P*<.001) and smoked for more years (*F*_1,502_=13.97; *P*<.001) than the participants who did not provide the training evaluation (age: mean 49.80, SD 11.76 years vs mean 43.95, SD 13.49 years; duration of years of smoking: mean 32.07, SD 12.54 vs mean 26.44, SD 13.64). Since there were no differences in TEQ responses between training versions for both AtBM and ApBM (results are reported in Multimedia Appendix 5), the overall responses to TEQs are summarized in Table 2. Among the 99 TEQ respondents, 49 quit the project during the training (ie, training dropouts) and 50 completed all 11 training sessions (ie, training completers). That is, 10.9% (49/450) of the training dropouts and 93% (50/54) of the training completers of this study evaluated the training. The TEQ responses for training dropouts and training completers are also separately summarized in Table 2. Compared with training completers, training dropouts were more negative on all the evaluation questions, thought the instructions of both training paradigms were less clear, and perceived both training interventions as less fun to do. Moreover, the 49 training dropouts also directly reported the reasons for their dropout. The top 4 reasons were as follows: 39% (19/49) of dropouts indicated that they were not satisfied with the training, 33% (16/49) of dropouts indicated that the training was too time consuming and they did not have time to do the training any more, 12% (6/49) of dropouts indicated that the training was boring, and 8% (4/49) of dropouts indicated that they thought they were in the sham training condition, which decreased their motivation to continue.

### Hypotheses Testing

The summary statistics of all outcomes by condition, time phase, and assessment time points are reported in Table 3. None of the outcome measures differed significantly across conditions at baseline (DCU: *F*_3,500_=0.79, *P*=.50; AtB: *F*_3,490_=0.33, *P*=.81; ApB: *F*_3,490_=1.26, *P*=.29). The results of the MLM analyses to test the training effects (omnibus effects) on all outcomes are reported in Table 4. The full MLM models for all outcomes can be found in Multimedia Appendix 6.

**Table 1:** Baseline characteristics of the final sample per condition.

Characteristics		Active-AtBM^a^ + active-ApBM^b^ (n=105)	Active-AtBM + sham-ApBM (n=132)	Sham-AtBM + active-ApBM (n=137)	Sham-AtBM + sham-ApBM (n=130)	*F* value (*df*_1_*,df*_2_)^c^ or chi-square value (*df*)^d^	*P* value
**Age (years)**						1.76 (3,500)^c^	.15
	Mean (SD)	44.98 (13.34)	46.86 (12.39)	45.38 (14.13)	43.10 (13.37)		
**Gender, n (%)**						7.9 (3)^d^	.048
	Male	30 (28.6)	34 (25.8)	54 (39.4)	49 (37.7)		
	Female	75 (71.4)	98 (74.2)	83 (60.6)	81 (62.3)		
**Highest education, n (%)**						5.7 (3)^d^	.13
	≥Bachelor’s degree	76 (72.4)	91 (68.9)	111 (81.0)	99 (76.2)		
	<Bachelor’s degree	29 (27.6)	41 (31.1)	26 (19.0)	31 (23.8)		
**Marital status, n (%)**						3.0 (3)^d^	.40
	Married	23 (21.9)	41 (31.1)	40 (29.2)	33 (25.4)		
	Other	82 (78.1)	91 (68.9)	97 (70.8)	97 (74.6)		
**Household income/month (**€**), n (%)**						0.8 (3)^d^	.86
	>3000	46 (43.8)	58 (43.9)	59 (43.1)	51 (39.2)		
	≤3000	59 (56.2)	74 (56.1)	78 (56.9)	79 (60.8)		
**Daily cigarette use in general**							
	Mean (SD)	16.15 (9.84)	18.32 (8.95)	17.23 (8.08)	16.12 (8.53)	1.77 (3,500)^c^	.15
**Duration of smoking (years)**							
	Mean (SD)	27.54 (13.16)	29.83 (12.77)	27.40 (14.31)	25.38 (13.81)	2.36 (3,500)^c^	.07
**mFTQ^e^** **(0 to 6)**							
	Mean (SD)	2.89 (1.73)	3.44 (1.53)	3.18 (1.58)	3.25 (1.63)	2.30 (3,500)^c^	.08
**Previous quit attempts**							
	Mean (SD)	5.82 (5.35)	5.54 (5.29)	5.89 (5.07)	5.55 (5.24)	0.16 (3,500)^c^	.93
**RCQ^f^** **(−24 to 24)**							
	Mean (SD)	12.62 (5.85)	11.85 (5.19)	11.80 (5.22)	12.26 (5.50)	0.59 (3,500)^c^	.62

^a^AtBM: attentional bias modification.

^b^ApBM: approach bias modification.

^c^One-way analyses of variance were conducted to test the baseline differences on continuous variables across the 4 conditions.

^d^Chi-square tests were conducted to test the baseline differences on categorical variables across the 4 conditions.

^e^mFTQ: Modified Fagerström Tolerance Questionnaire.

^f^RCQ: Readiness to Change Questionnaire.

**Table 2:** Summary of training evaluation responses.

Training evaluation questions		TEQ^a^ respondents (n=99)	Training dropouts (n=49)	Training completers (n=50)	*F* value (*df*_1_*,df*_2_)^b^ or chi-square value (*df*)^c^	*P* value
**Training evaluation for CBM^d^** **as a whole**						
	1. What do you think of the quality of this CBM training?^e^, n (%)	47 (47.5)	15 (30.6)	32 (64.0)	11.1 (1)^c^	.001
	2. How satisfied are you overall with this CBM training?^f^, n (%)	47 (47.5)	16 (32.7)	31 (62.0)	8.6 (1)^c^	.003
	3. I think the CBM training helped me with my problems^g^, mean (SD)	3.43 (1.99)	2.73 (1.63)	4.12 (2.08)	13.60 (1,97)^b^	<.001
	4. Would you recommend this CBM training to others?^h^, n (%)	48 (48.5)	16 (32.7)	32 (64.0)	9.7 (1)^c^	.002
	5. Will you use the CBM training in the further?^h^, n (%)	65 (65.7)	23 (46.9)	42 (84.0)	15.1 (1)^c^	<.001
**Training evaluation for AtBM^i^**						
	1. The goal of the AtBM training was clear before I started it^g^, mean (SD)	4.98 (1.84)	4.69 (2.06)	5.26 (1.56)	2.37 (1,97)^b^	.13
	2. The instructions on what I should do during the AtBM training was clear^g^, mean (SD)	6.05 (1.55)	5.73 (1.80)	6.36 (1.21)	4.13 (1,97)^b^	.045
	3. The AtBM training was difficult to do^g^, mean (SD)	3.25 (1.83)	3.53 (1.89)	2.98 (1.74)	2.27 (1,97)^b^	.14
	4. The AtBM training was fun to do^g^, mean (SD)	3.29 (1.93)	2.61 (1.74)	3.96 (1.88)	13.65 (1,97)^b^	<.001
**Training evaluation for ApBM^j^**						
	1. The goal of the ApBM training was clear before I started it^g^, mean (SD)	5.11 (1.88)	4.80 (2.04)	5.42 (1.67)	2.78 (1,97)^b^	.10
	2. The instructions on what I should do during the ApBM training was clear^g^, mean (SD)	5.98 (1.48)	5.65 (1.79)	6.30 (1.02)	4.94 (1,97)^b^	.03
	3. The ApBM training was difficult to do^g^, mean (SD)	2.89 (1.80)	3.18 (1.83)	2.60 (1.73)	2.66 (1,97)^b^	.11
	4. The ApBM training was fun to do^g^, mean (SD)	3.81 (2.07)	3.02 (1.96)	4.58 (1.89)	16.26 (1,97)^b^	<.001

^a^TEQ: training evaluation question.

^b^One-way analyses of variance were conducted to test the differences in modal responses of the training evaluation between training dropouts and training completers.

^c^Chi-square tests were conducted to test the differences in average responses of the training evaluation between training dropouts and training completers.

^d^CBM: cognitive bias modification.

^e^Poor, fair, good, excellent; percentage of “good” and “excellent” responses.

^f^Very dissatisfied, fairly dissatisfied, fairly satisfied, very satisfied; percentage of “fairly satisfied” and “very satisfied” responses.

^g^Participants indicated the extent to which they agreed with this statement on a scale from 1 (completely disagree) to 7 (completely agree).

^h^No, definitely not; No, I do not think so; Yes, I think so; Yes, definitely; percentage of “Yes, I think so” and “Yes, definitely” responses.

^i^AtBM: attentional bias modification.

^j^ApBM: approach bias modification.

**Table 3:** Summary statistics on outcomes by condition, time phase, and assessment time points.

Outcomes, time phase, and assessment time points			Active-AtBM^a^ + active-ApBM^b^	Active-AtBM + sham-ApBM	Sham-AtBM + active-ApBM	Sham-AtBM + sham-ApBM
**PPA^c^, n_1_/n_2_ (%)^d^**						
		Baseline	0/105 (0)	0/132 (0)	0/137 (0)	0/130 (0)
		IA^e^1	11/79 (14)	16/111 (14.4)	5/110 (4.5)	8/94 (9)
		IA2	7/51 (14)	9/70 (13)	5/75 (7)	11/61 (18)
		IA3	7/34 (21)	6/39 (15)	6/54 (11)	9/49 (18)
		IA4	5/28 (18)	6/33 (18)	7/43 (16)	5/34 (15)
		IA5	8/23 (35)	4/27 (15)	8/33 (24)	3/26 (12)
		Mid^f^	7/21 (33)	2/24 (8)	6/27 (22)	3/20 (15)
	*TP1^g^*		*45/341 (13.2)*	*43/436 (9.9)*	*37/479 (7.7)*	*39/414 (9.4)*
		IA6	8/19 (42)	4/21 (19)	5/24 (21)	2/20 (10)
		IA7	7/17 (41)	2/19 (11)	4/20 (20)	3/19 (16)
		IA8	6/17 (35)	3/17 (18)	2/19 (11)	3/19 (16)
		IA9	5/15 (33)	2/14 (14)	3/15 (20)	3/17 (18)
		IA10	5/14 (36)	2/13 (15)	3/12 (25)	2/15 (13)
		Post^h^	5/14 (36)	2/11 (18)	3/10 (30)	4/15 (27)
	*TP2^i^*		*36/96 (38)*	*15/95 (16)*	*20/100 (20.0)*	*17/105 (16.2)*
		IA11	5/13 (38)	2/11 (18)	3/10 (30)	4/15 (27)
		FU1^j^	5/13 (39)	3/11 (27)	3/10 (30)	1/14 (7)
		FU2^k^	3/12 (25)	3/11 (27)	3/9 (33)	1/13 (8)
		FU3^l^	4/12 (33)	2/9 (22)	3/9 (33)	2/12 (17)
	*TP3^m^*		*17/50 (34)*	*10/42 (24)*	*12/38 (32)*	*8/54 (15)*
**DCU^n^, mean (SD)^o^**						
	*TP1 (baseline)*		*15.25 (9.70)*	*16.71 (9.97)*	*15.43 (8.48)*	*15.20 (8.44)*
		IA1	11.23 (8.60)	12.96 (10.58)	12.89 (8.22)	13.48 (8.98)
		IA2	11.07 (9.11)	13.07 (10.12)	12.48 (8.63)	10.97 (9.76)
		IA3	10.41 (9.99)	13.30 (10.48)	11.96 (9.23)	9.74 (8.46)
		IA4	10.06 (10.71)	13.15 (11.09)	11.39 (9.93)	11.36 (8.93)
		IA5	9.26 (9.54)	14.15 (11.30)	11.58 (10.73)	9.24 (8.28)
		Mid	7.77 (10.25)	15.14 (9.94)	12.52 (10.94)	11.55 (11.36)
	*TP2*		*10.44 (9.38)*	*13.33 (10.47)*	*12.31 (9.13)*	*11.52 (9.23)*
		IA6	6.79 (9.27)	14.71 (10.37)	13.15 (11.74)	10.33 (9.68)
		IA7	7.84 (10.36)	15.13 (9.60)	13.70 (11.71)	10.62 (9.94)
		IA8	8.49 (10.37)	15.15 (10.74)	13.15 (12.25)	10.19 (10.22)
		IA9	8.56 (10.21)	15.24 (11.53)	12.10 (11.63)	8.68 (7.59)
		IA10	10.16 (10.65)	14.53 (10.03)	11.48 (10.75)	7.14 (6.99)
		Post	8.67 (10.45)	14.63 (10.79)	9.22 (8.93)	7.48 (7.72)
	*TP3*		*8.32 (9.97)*	*14.92 (10.19)*	*12.51 (11.25)*	*9.22 (8.81)*
		IA11	10.56 (12.26)	14.07 (10.98)	9.15 (8.10)	7.00 (7.25)
		FU1	11.70 (16.09)	15.77 (14.69)	7.99 (8.53)	6.55 (6.19)
		FU2	12.21 (14.00)	13.42 (14.98)	8.27 (9.78)	8.23 (6.20)
		FU3	12.29 (13.19)	15.68 (18.99)	8.83 (9.05)	8.70 (7.23)
	*TP4^p^*		*11.67 (13.55)*	*14.69 (14.43)*	*8.56 (8.50)*	*7.56 (6.60)*
**AtB^q^, mean (SD)^o^**						
	*TP1 (baseline)*		*24.15 (30.69)*	*27.42 (27.65)*	*24.84 (27.36)*	*24.56 (29.79)*
		IA1	23.06 (37.74)	16.36 (36.17)	27.27 (40.80)	23.95 (28.87)
		IA2	15.32 (42.95)	15.92 (32.99)	18.10 (35.52)	15.85 (31.78)
		IA3	15.42 (32.93)	5.62 (46.13)	19.84 (37.84)	21.73 (39.93)
		IA4	6.89 (33.65)	−2.38 (32.61)	20.40 (43.01)	23.65 (35.33)
		IA5	−0.88 (20.32)	−1.86 (32.68)	8.92 (53.41)	14.02 (30.31)
		Mid	3.40 (33.38)	8.35 (25.93)	3.29 (25.17)	16.45 (18.61)
	*TP2*		*14.05 (36.44)*	*10.00 (36.04)*	*19.39 (40.38)*	*20.20 (31.98)*
		IA6	15.21 (29.28)	9.78 (34.79)	12.41 (40.02)	14.28 (24.70)
		IA7	11.24 (26.26)	0.92 (28.94)	4.65 (31.91)	16.16 (28.66)
		IA8	14.24 (32.64)	5.44 (31.32)	5.64 (34.85)	9.17 (29.67)
		IA9	4.33 (43.25)	4.89 (27.51)	5.50 (31.64)	8.32 (30.34)
		IA10	4.71 (33.25)	−2.00 (32.02)	−4.42 (42.26)	8.50 (17.07)
		Post	−4.08 (12.82)	−2.46 (28.18)	10.70 (25.76)	2.47 (26.12)
	*TP3*		*8.42 (30.96)*	*3.35 (30.33)*	*6.23 (34.72)*	*10.23 (26.39)*
		IA11	1.19 (32.46)	−16.59 (23.64)	−2.15 (25.38)	5.67 (33.41)
		FU3	7.36 (14.38)	2.28 (16.09)	4.25 (23.70)	8.96 (17.65)
	*TP4*		*4.02 (25.49)*	−*8.10 (22.27)*	*0.69 (24.14)*	*7.13 (27.12)*
**ApB^r^, mean (SD)^o^**						
	*TP1 (baseline)*		−*7.84 (65.28)*	*8.82 (84.61)*	*4.52 (67.04)*	*9.70 (77.34)*
		IA1	4.88 (77.67)	−11.23 (114.00)	11.25 (113.95)	4.66 (98.25)
		IA2	7.10 (76.98)	−7.74 (71.96)	−0.17 (78.86)	9.56 (82.04)
		IA3	1.15 (73.75)	−4.47 (46.16)	−1.83 (82.75)	12.24 (59.45)
		IA4	−35.76 (42.72)	12.82 (70.46)	10.87 (75.61)	9.80 (71.82)
		IA5	−2.50 (73.86)	−13.05 (53.32)	0.79 (62.30)	−7.06 (68.51)
		Mid	7.85 (47.22)	−6.87 (27.64)	−10.35 (91.38)	11.30 (48.89)
	*TP2*		−*0.50 (71.27)*	−*6.47 (81.99)*	*3.72 (90.65)*	*7.04 (79.30)*
		IA6	−12.18 (56.42)	8.10 (54.18)	−26.52 (99.34)	4.58 (41.78)
		IA7	−10.68 (44.52)	18.58 (47.19)	−3.10 (67.67)	3.97 (99.19)
		IA8	6.94 (49.90)	−2.09 (49.79)	26.66 (82.69)	8.47 (47.50)
		IA9	−7.70 (78.92)	−1.46 (42.34)	−5.10 (31.09)	−3.12 (62.89)
		IA10	−22.75 (38.49)	20.50 (45.27)	−21.67 (39.40)	−9.87 (53.18)
		Post	−17.96 (35.54)	26.58 (32.70)	−5.60 (36.41)	−13.10 (29.10)
	*TP3*		−*10.13 (52.45)*	*10.99 (46.77)*	−*5.42 (71.26)*	−*0.75 (59.90)*
		IA11	−14.96 (52.63)	20.36 (51.52)	−15.70 (22.51)	34.20 (66.24)
		FU3	2.27 (41.97)	−4.39 (41.06)	2.06 (54.69)	−12.04 (59.54)
	*TP4*		−*7.06 (47.83)*	*9.22 (47.61)*	−*7.81 (39.78)*	*13.65 (66.41)*

^a^AtBM: attentional bias modification.

^b^ApBM: approach bias modification.

^c^PPA: point prevalence abstinence.

^d^For PPA at each assessment time point, n_1_/n_2_= number of participants coded as 1 (that is, quit) at that assessment time point divided by the total number of participants who reported their smoking status at that assessment time point; at each time phase, n_1_/n_2_= sum of observations coded as 1 (that is, quit) at that time phase divided by sum of all reported observations at that time phase.

^e^IA: interim assessment.

^f^Mid: midtraining assessment.

^g^TP1: time phase 1, first half intervention phase for PPA; baseline for DCU, AtB, and ApB.

^h^Post: posttraining assessment.

^i^TP2: time phase 2, second half intervention phase for PPA, first half intervention phase for DCU, AtB, and ApB.

^j^FU1: follow-up assessment at 1 month.

^k^FU2: follow-up assessment at 2 months.

^l^FU3: follow-up assessment at 3 months.

^m^TP3: time phase 3, follow-up phase for PPA; second half intervention phase for DCU, AtB, and ApB.

^n^DCU: daily cigarette use.

^o^For DCU, AtB, and ApB, at each assessment time point, average score at the assessment time point is reported; at each time phase, time average score at that time phase is reported.

^p^TP4: time phase 4, follow-up phase for DCU, AtB, and ApB.

^q^AtB: attentional bias for smoking stimuli.

^r^ApB: approach bias for smoking stimuli.

**Table 4:** Results of multilevel modeling analyses.

Omnibus effects	PPA^a^		DCU^b^		AtB^c^		ApB^d^	
	Chi-square value (*df*)	*P* value	*F* value (*df*_1_*,df*_2_)	*P* value	*F* value (*df*_1_*,df*_2_)	*P* value	*F* value (*df*_1_*,df*_2_)	*P* value
TP^e^	3.7 (2)	.15	111.98 (3,1835.75)	<.001	17.62 (3, 1930.01)	<.001	0.34 (3,2049)	.80
AtBM^f^	0.0 (1)	.90	0.04 (1,620.77)	.84	0.31 (1,766.99)	.58	0.48 (1,2049)	.49
ApBM^g^	0.5 (1)	.49	2.64 (1,620.77)	.11	0.01 (1,766.99)	.91	4.43 (1,2049)	.04
TP × AtBM	3.6 (2)	.17	1.34 (3,1835.75)	.26	2.35 (3,1930.01)	.07	0.67 (3,2049)	.57
TP × ApBM	1.2 (2)	.56	2.23 (3,1835.75)	.08	0.32 (3,1930.01)	.81	1.47 (3,2049)	.22
AtBM × ApBM	0.4 (1)	.50	1.82 (1,620.77)	.18	3.62 (1,766.99)	.06	0.13 (1,2049)	.73
TP × AtBM × ApBM	4.0 (2)	.14	1.86 (3,1835.75)	.13	1.78 (3,1930.01)	.15	0.99 (3,2049)	.40

^a^PPA: point prevalence abstinence.

^b^DCU: daily cigarette use.

^c^AtB: attentional bias for smoking stimuli.

^d^ApB: approach bias for smoking stimuli.

^e^TP: Time phase.

^f^AtBM: attentional bias modification.

^g^ApBM: approach bias modification.

#### Primary Outcome

With respect to PPA, no significant effects emerged from the MLM analysis (Table 4), indicating that neither training versions nor their combination had a significant impact on PPA over time. However, note that descriptively, the double active training condition showed the highest PPA rate at each time phase of the study (Table 3).

#### Secondary Behavioral Outcome

With respect to DCU, the MLM analysis only indicated a significant main effect of time phase (Table 4). From baseline to the first half of the intervention, all participants had a significant reduction in DCU (*B*=−3.89, 95% CI −4.96 to −2.83; *P*<.001; *d*=0.43), regardless of training condition. This effect persisted to the second half of the intervention (*B*=-5.46, 95% CI −6.91 to −4.00; *P*<.001; *d*=0.60) and to the follow-ups (*B*=-4.61, 95% CI −6.32 to −2.90; *P*<.001; *d*=0.50). Contrary to our hypotheses, no significant two- or three-way interaction effects between training version and time phase emerged, suggesting that neither training versions nor their combination had a significant impact on DCU over time.

#### Secondary Cognitive Outcomes

At baseline, overall, participants demonstrated an AtB toward smoking-related stimuli (mean 25.30, SD 28.73; t_493_=19.57; *P*<.001), but demonstrated neither an approach nor an avoidance bias (mean 4.43, SD 74.43; t_492_=1.32; *P*=.18). Baseline AtB was positively correlated with the duration of years of smoking (*r*=0.21, *P*<.001), while baseline ApB was positively correlated to nicotine dependence (*r*=0.11, *P*=.01) and DCU in general (*r*=0.12, *P*=.01). The two biases were not correlated with each other at baseline (*r*=0.06, *P*=.20).

The MLM analysis only indicated a main effect of time phase on AtB (Table 4). All participants showed a significant reduction in AtB from baseline to the second half of the intervention (*B*=−10.28, 95% CI −18.98 to −1.58; *P*=.02; *d*=0.36), but this reduction did not maintain up to the 3-month follow-up (*B*=−11.03, 95% CI −24.05 to 1.99; *P*=.10, *d*=0.38). Although there was also a main effect of ApBM on ApB (Table 4), follow-up analyses showed that none of the regression coefficients involving ApBM was significant (Multimedia Appendix 6). Contrary to our hypotheses, there were no significant interaction effects between training version and time phase on both AtB and ApB. This suggests that both the AtBM and ApBM did not affect the respective cognitive bias it targeted over time.

### Summary of Additional Analyses

The methods and results for testing training effects on the additional secondary outcomes (ie, craving, depression severity, and motivation to quit smoking) and for the exploratory moderation analysis on participants’ awareness of CBM condition are reported in Multimedia Appendices 1 and 2. The main findings were (1) all participants showed a significant reduction in craving over time, and no training effects on depression severity and motivation to quit smoking were observed; (2) 19.6% (99/504) of the final sample (ie, TEQ respondents) indicated their awareness of training condition, the majority of whom thought they completed the sham training, while they actually completed the active one, for both training types; and (3) participants’ awareness of CBM condition moderated training effects in DCU: participants who correctly thought that they were in the active training condition (for either training type) showed larger decreases in DCU over time compared with those who thought they were in the sham training condition but actually completed the active training.

## Discussion

### Principal Findings

This double-blind RCT tested the individual and combined effects of web-based AtBM and ApBM in adult smokers seeking online help for quitting smoking. Against our expectations, we did not find evidence for the effectiveness of neither CBM trainings nor their combination, compared with their respective sham version, in improving any of the smoking-related outcomes. In addition, neither did any of the CBM training conditions change the targeted cognitive biases. Compliance to the intervention was very low as only 10.7% (54/504) of participants completed all training sessions, and 8.3% (42/504) of the participants completed the follow-up assessments, suggesting that the web-based intervention was not well accepted.

The results indicated a general improvement in smoking-related behaviors irrespective of condition. That is, participants in all conditions may have tried to quit smoking and reduced their DCU over time. The enrollment in the intervention is a sign for motivation to change, which may suggest that the training did not have an effect larger than the mere motivation for participants to do something about their behavior and enroll in a self-help web-based program. This general improvement might also be attributable to features of the intervention that were common to all participants, including exposure to automated tailored feedback and self-monitoring of smoking behaviors. In addition, the general improvement is also likely to be driven by the high dropout rates. That is, those who stayed in the intervention longer may have produced a floor effect because of a greater self-confidence and motivation to change their smoking behaviors or a better ability to master their smoking behaviors.

The null findings on the smoking-related outcomes are consistent with recent studies examining web-based CBM in smokers [11,28] and in problem drinkers [15,53,54], except for one study using a waitlist (passive control) instead of the sham training (active control) as a comparator condition [27]. However, our findings are at odds with the studies examining CBM effects in the clinical setting with inpatient psychiatric smokers [29] and alcohol-dependent inpatients [17,18,55,56], except for 2 studies with a much smaller sample size [31,32]. Therefore, differences between the 2 types of studies, online and in the clinic, may explain the inconsistent findings [15].

A major difference is that CBM is normally administrated as an add-on intervention to the standard treatment in the clinical setting (eg, CBT [17,18,28,29]), whereas as a stand-alone or primary intervention in the online setting (as in this study). Although automated tailored feedback was included as a cointervention in this study, it was minimal and its effectiveness might have been threatened by its static feature. It is possible that CBM interventions only produce effects when blended with other standard treatments targeting more controlled cognitive processes aimed at long-term health outcomes. A related difference is that, in a clinical setting, CBM is administrated in a guided environment (ie, with the support of the therapists), while an unguided environment online fully relies on participants’ autonomy, self-reliance, and self-discipline [57]. The lack of personal contact or therapist-client interaction in an online setting may increase the feelings of lack of support. Indeed, effects of web-based interventions can be enhanced when brief face-to-face communication with therapists [58] or various forms of remote support from therapists (eg, emails or telephone calls [59]) are included. However, accessing in-person standard treatment and support from therapists in a blended format would cost time and money, thereby limiting the feasibility of the widespread implementation and cost-effectiveness of online CBM. Thus, future online CBM studies may benefit by incorporating online CBM training with online standard treatment and additional remote support from therapists as a treatment package. This new design, building on the previous studies in a clinical context may also increase the credibility of online CBM training (a topic further discussed below).

In addition, compared with clinical settings, participants recruited online are more heterogeneous in terms of severity of symptoms. In this study, the CBM training program was open to any adult smoker seeking online help to quit smoking. As a result, our sample was very diverse in terms of severity of tobacco dependence resulting in mild smoking problems on average. Considering that CBM overall has shown small effects as an adjunct intervention in clinical settings with severely addicted patients [15,26], to find a similar effect size in such a heterogeneous population of interest, the sample size would need to be much larger [60], especially to also account for the higher number of dropouts typical of unguided online interventions.

Aside from a nonspecific improvement in smoking-related outcomes, none of the targeted cognitive biases were influenced by the 2 variants of CBM. This is in line with few previous studies evaluating CBM as a smoking behavior change intervention, which hardly found any evidence of specific reductions in the targeted cognitive bias online [11,28] and in clinical or laboratory settings [29-32]. There is one exception [33], although its interpretation is complicated by a very different experimental design (3 sessions of active AtBM training compared with 1 session of sham training). Presumably, CBM interventions would show substantial effects on behaviors once the targeted mechanism of change (changing the targeted bias) is successfully engaged, which so far has not been the case. Furthermore, at baseline, we only found a moderate AtB toward smoking-related stimuli, and no ApB, suggesting little room for CBM training effects.

Recently, there has been a debate about the optimal comparison condition in CBM studies [61,62]. In the standard sham training condition (as in this study), participants learn to shift attention away or avoid smoking stimuli in half of the trials, which is very similar to the active training condition and may leave no room for the active training to produce specific effects. A recent Bayesian meta-analysis of clinical CBM studies in addiction found larger training effects in the control condition involving mostly sham training, relative to the active training condition, with an increased dosage of training [26]. This might point to a slower learning mechanism (perhaps exposure) in addition to a quicker and short-lasting change in bias in the active condition. Therefore, we may need to make larger differences between the active and sham versions of the training to detect specific effects of CBM. To specifically evaluate clinical effects of CBM, one solution could be to carefully choose a more appropriate control condition, and another solution could be to make active training more meaningful to participants, for example, by training participants to approach (personalized) meaningful stimuli rather than neutral stimuli in addition to training them to avoid smoking stimuli [63] or by providing positive or relevant rewards to reinforce newly learned behaviors (eg, avoid smoking stimuli) during training [64].

### Limitations and Future Research

The most notable limitation of this study is the low training adherence and the high dropout rates. Although high dropout rates are similar to the few published web-based CBM studies [54,65] and are very common in online interventions [60,66,67], this issue may have limited the validity of the results and caused power issues in this study.

A second important limitation that may have affected the low degree of engagement and adherence with the intention concerns the *top-down* approach we used to design this intervention. We developed this intervention by using a *theory and evidence-based* approach; therefore, we moved the typical CBM trainings delivered in the clinical setting to the online setting, by considering the online environment as a mere *delivery box* to a larger public and not as a new component of the intervention likely affecting its reception. Furthermore, since we did not incorporate any potential users’ perspective or feedback into the intervention design, the intervention may not have met the users’ needs and preferences (ie, the program was not user-friendly and engaging enough [57,68]). These hypotheses were supported by the training evaluation provided by a minority of participants (ie, the TEQ respondents: 19.6% (99/504) of the final sample). It should be noted that the TEQ respondents were older and smoked for more years than those who did not provide the training evaluation; therefore, they are not representative of the whole sample. For this reason, these findings should be interpreted with some caution. In addition, to obtain a more representative sample, especially those who left the intervention without providing an evaluation of the intervention should be approached. Future research could adopt the strategy of monetary incentives to increase these response rates [69] or make more effort to interview the dropouts to understand their needs and thoughts to improve the intervention.

Although the TEQ respondents in this study are not representatives of the whole sample, their feedback can be very valuable in pinpointing factors contributing to the lack of success of the study. For example, as a reason to leave the intervention, some training dropouts reported that the training was too time consuming. Indeed, this study included a lot of assessments before and during the intervention, which may have increased the burden on the participants. Although repeatedly measuring the cognitive biases during the training allows to study progressive changes, this setting may have taxed participants’ motivation to train and may also have interfered with the training effects [17,18]. Therefore, a recommendation for future research is to keep the amount of measurements to a sufficient minimum.

In addition, compared with training completers, training dropouts indicated the training was less fun to do, and some of them explicitly reported that it was boring. Indeed, the CBM tasks had an intrinsic repetitive nature. To improve the motivation to train and compliance, future research is recommended to make the CBM task more interesting and engaging by, for example, gamifying it [24,70]. Besides the dullness of the CBM tasks, the intervention website had a very simple layout and only provided text-based information to the participants. Participants were required to read and process much information to understand the study and its procedure, without an alternative source of information, such as video or graphics. The amount and length of the text may have challenged participants’ literacy level and attention span and led participants to become overwhelmed or bored (compare Atkinson et al [71]).

Moreover, compared with training completers, training dropouts indicated that the training instructions were less clear, and some of them also explicitly reported that they perceived that they received the sham training and were therefore likely demotivated to continue. In this study, we used an indirect version of CBM training, where participants are required to respond to an irrelevant feature of the stimuli (eg, orientation of the probe in AtBM and tilted format of the stimuli in ApBM) rather than the content of the stimuli (ie, smoking-related or neutral stimuli). As a result, we have replicated the results of previous research that a majority of the participants in both training conditions believed that they were in the sham training condition [72]. This is positive from a blinding to conditions perspective, but suboptimal from a motivational clinical perspective. In general, with indirect instructions, participants often have difficulties understanding how the training is relevant to their problem, which may threaten the credibility of the training [72] and may have led them to feel disappointed when perceiving that they were assigned to a sham treatment [73]. In addition, our exploratory moderation analyses of awareness of training version (Multimedia Appendix 2) showed that participants who correctly thought that they were in the active training condition for either training type, showed a larger decrease in DCU over time compared with those who thought they were in the sham training condition but they actually completed the active training. It should be noted that these results need to be interpreted with caution given that the exploratory moderation analyses were conducted on a minority of participants and the mechanism of the moderation effects was unclear, since participants were only asked about what training version they perceived they received rather than the exact contingencies between stimuli and their responses. Yet, this information may also point to the importance of providing explicit and clear task instructions to improve the intervention credibility (see also Van Dessel et al [74,75]). Furthermore, the credibility of the online CBM training may also have been threatened since participants were informed that they had a 25% chance to be assigned to a condition combining two sham trainings (where smaller or no effects were expected), likely affecting their compliance to and acceptance of the intervention. This limitation is inevitable since this information should be provided to meet ethical standards [76].

A last notable limitation refers to the unsatisfactory reliabilities for both the VPT and AAT in this study, consistently with most implicit tasks [77]. As a result, it still remains unsolved whether the CBM intervention did not change the cognitive biases or whether we were merely not able to assess any changes in the biases reliably. Therefore, it is necessary to develop more reliable experimental tasks for measuring cognitive biases in further research.

### Conclusions

This was the first study to evaluate whether combining 2 CBM paradigms was effective as a self-help web-based intervention for smoking cessation. Contrary to our hypotheses, the results only revealed a general reduction in DCU across time in all conditions, suggesting no beneficial effects that can be directly attributed to any of the web-based CBM training or their combination. The study had very high dropout rates and a very low frequency of training usage, indicating an overall low acceptability of the intervention, which precludes any definite conclusion on its effectiveness. Before drawing firm conclusions regarding the effectiveness of online CBM training in smokers, a fully powered study with a more engaging version of smoking CBM in a large sample is needed. Therefore, further studies on online CBM should improve the intervention compliance and prevent dropouts as a first step, whereas the overall design of the next online CBM intervention would benefit greatly from being not only *theory and evidence-based* but also *user-centered* to ensure engagement and retainment by its users. In addition, to translate findings on CBM in clinical settings into a viable and effective behavior change intervention in the real world, substantial modification of the training procedure and core design is needed.

## Appendix

Multimedia Appendix 1 Additional secondary outcomes.

Multimedia Appendix 2 Exploratory moderation analysis.

Multimedia Appendix 3 Task stimuli.

Multimedia Appendix 4 Task data preparation.

Multimedia Appendix 5 Summary of training evaluation responses in the training condition of attentional bias modification and approach bias modification, respectively.

Multimedia Appendix 6 Full multilevel models for primary and secondary outcomes.

Multimedia Appendix 7 CONSORT EHEALTH checklist (V1.6.1).

## Abbreviations

AAT: approach-avoidance task
ADAPT: Addiction Development and Psychopathology Lab of the University of Amsterdam
ApB: approach bias
ApBM: approach bias modification
AtB: attentional bias
AtBM: attentional bias modification
CBM: cognitive bias modification
CBT: cognitive behavioral therapy
DCU: daily cigarette use
ITT: intention-to-treat
mFTQ: Modified Fagerström Tolerance Questionnaire
MLM: multilevel modeling
PPA: point prevalence abstinence
RCQ: Readiness to Change Questionnaire
RCT: randomized controlled trial
TEQ: training evaluation question
TP: time phase
VPT: visual probe task
