# Supplementary material for: Combining Web-Based Attentional Bias Modification and Approach Bias Modification as a Self-Help Smoking Intervention for Adult Smokers Seeking Online Help: Double-Blind Randomized Controlled Trial
Source: JMIR Ment Health. 2020 May 8;7(5):e16342. doi: 10.2196/16342 (PMC7244992; doi:10.2196/16342)
Supplement: Multimedia Appendix 1 [file mental_v7i5e16342_app1.doc]

## Multimedia Appendix 1. Additional secondary outcomes

Since craving, depression severity, and motivation to quit smoking have been found to be related to cognitive biases or smoking behaviors [1-3], these variables were also included in this study as additional secondary outcomes.

### Methods

Craving and depression severity were measured during six main assessments (i.e., at baseline, mid-training, post-training, 1-, 2-, and 3-month follow-ups). Motivation to quit smoking was measured at the start of each training session (i.e., 11 interim assessments).

Craving was measured with the 10-item Questionnaire on Smoking Urges (QSU-Brief, [4]), which has a total score ranging from 10 to 70 (low to high level of craving). The internal consistency (Cronbach’s α) for QSU at baseline was .93. Depression severity was measured using the 7-item Beck Depression Inventory Fast Screen (BDI-FS, [5]), with a sum score ranging from 0 to 21 (low to high level of depression). The internal consistency (Cronbach’s α) for BDI at baseline was .82. Motivation to quit (MTQ) was measured with a Visual Analogue Scales (VAS). Participants indicated to what extent they were currently motivated to quit smoking on a scale from 0 (‘not motivated at all’) to 100 (‘very motivated).

Training effects on the additional secondary outcomes were analyzed similar to the main primary and secondary outcomes, except for the coding of the Time or Time Phase predictor. For QSU and BDI, the training effects were evaluated at four end-points: baseline (T1), mid-training assessment (T2), post-training assessment (T3), and follow-up phase (T4: from 1- to 3-month follow-up). For MTQ, the training effects were evaluated at three Time Phases of the study: baseline (T1: interim assessment 1), first half of the intervention (TP2: interim assessment 2 to 6), and second half intervention phase (TP3: from interim assessment 7 to11). Holm-Bonferroni correction was applied to all analyses to account for multiple testing.

### Results

The summary statistics for the additional secondary outcomes by condition, time phase and assessment time-points are reported in Table S1. None of the measures differed significantly across conditions at baseline (QSU: *F*(3, 500) = 1.71, *P* = .17; BDI: *F*(3, 500) = 0.94, *P* = .42; MTQ: *F*(3, 394) = 1.00, *P* = .39). The results of the MLM analyses to test training effects (omnibus effects) on the additional secondary outcomes are reported in Table S2. The full MLM models for all the additional secondary outcomes can be found from Table S3 to Table S5.

**Table S1.** Summary statistics on additional secondary outcomes by Condition, Time/Time Phase, and/or

Assessment time-points

| Outcomes^a^ | Time/Time Phase^b^ | Assessment time-points^c^ | active-AtBM +  active-ApBM^d^ | active-AtBM +  sham-ApBM | sham-AtBM +  active-ApBM | sham-AtBM +  sham-ApBM |
| --- | --- | --- | --- | --- | --- | --- |
| **QSU**  ***M* (*SD*)^e^** | **T1** | **Baseline** | **33.84 (15.57)** | **35.59 (15.39)** | **34.19 (15.16)** | **31.40 (15.03)** |
|  | **T2** | **Mid** | **28.71 (11.15)** | **32.83 (13.47)** | **30.81 (15.68)** | **24.40 (12.99)** |
|  | **T3** | **Post** | **29.36 (11.97)** | **28.92 (16.37)** | **21.70 (8.49)** | **22.73 (10.30)** |
|  |  | FU1 | 30.08 (14.76) | 24.36 (17.65) | 22.70 (13.14) | 22.57 (9.79) |
|  |  | FU2 | 26.92 (15.93) | 25.00 (13.17) | 24.44 (13.82) | 27.92 (14.52) |
|  |  | FU3 | 31.50 (14.37) | 22.11 (11.83) | 25.56 (13.54) | 29.58 (15.72) |
|  | **T4** |  | **29.51 (14.73)** | **23.94 (14.16)** | **24.18 (13.04)** | **26.51 (13.43)** |
| **BDI**  ***M* (*SD*)** | **T1** | **Baseline** | **3.47 (3.29)** | **4.20 (3.69)** | **3.97 (3.49)** | **3.79 (3.39)** |
|  | **T2** | **Mid** | **2.62 (2.56)** | **4.42 (3.68)** | **2.44 (2.61)** | **4.35 (4.73)** |
|  | **T3** | **Post** | **2.14 (2.32)** | **3.42 (3.34)** | **2.30 (1.89)** | **4.33 (4.95)** |
|  |  |  |  |  |  |  |
|  |  | FU1 | 3.00 (4.26) | 3.55 (3.27) | 1.90 (2.18) | 4.21 (4.04) |
|  |  | FU2 | 2.33 (3.45) | 4.18 (5.17) | 1.89 (2.15) | 2.62 (2.60) |
|  |  | FU3 | 2.00 (3.79) | 3.44 (4.36) | 1.44 (1.88) | 4.92 (2.84) |
|  | **T4** |  | **2.46 (3.78)** | **3.74 (4.20)** | **1.75 (2.01)** | **3.90 (3.31)** |
| **MTQ**  ***M* (*SD*)** | **TP1** | **IA1** | **70.74 (26.75)** | **66.40 (26.83)** | **70.68 (24.22)** | **65.88 (27.06)** |
|  |  | IA2 | 68.61 (26.50) | 65.23 (24.32) | 67.97 (23.90) | 68.90 (27.22) |
|  |  | IA3 | 73.06 (26.23) | 67.00 (27.29) | 71.25 (28.32) | 71.65 (25.46) |
|  |  | IA4 | 75.93 (23.55) | 68.24 (26.54) | 70.07 (26.71) | 68.35 (30.32) |
|  |  | IA5 | 72.96 (27.20) | 69.61 (24.17) | 71.06 (31.58) | 75.23 (26.04) |
|  |  | IA6 | 76.05 (22.91) | 68.71 (23.92) | 71.12 (28.54) | 72.55 (26.49) |
|  | **TP2** |  | **72.47 (25.47)** | **67.12 (25.06)** | **69.91 (26.97)** | **70.74 (26.95)** |
|  |  | IA7 | 72.00 (26.17) | 63.21 (26.50) | 71.70 (30.31) | 63.47 (32.43) |
|  |  | IA8 | 71.59 (28.77) | 64.65 (25.31) | 75.16 (26.89) | 65.26 (27.72) |
|  |  | IA9 | 77.47 (18.64) | 67.93 (19.40) | 80.00 (24.78) | 66.71 (29.57) |
|  |  | IA10 | 66.57 (29.18) | 55.79 (22.36) | 86.83 (21.02) | 79.87 (18.50) |
|  |  | IA11 | 71.15 (22.94) | 63.45 (28.61) | 74.30 (32.35) | 83.67 (18.24) |
|  | **TP3** |  | **71.84 (25.12)** | **63.07 (24.27)** | **76.93 (27.16)** | **70.98 (27.13)** |

^a^QSU: Questionnaire on Smoking Urges; BDI: Beck Depression Inventory Fast Screen; MTQ: Motivation to quit smoking.

^b^Time: T1 (baseline), T2 (mid-assessment), T3 (post-assessment), T4 (follow-up phase) for QSU and BDI; Time Phase: TP1 (baseline), TP2 (first half intervention phase), TP3 (second half intervention phase) for MTQ.

^c^IA1 to IA11: interim assessment 1 to 11; Mid: mid-training assessment; Post: post-training assessment; FU1, FU2, FU3: follow-up assessment at 1, 2, and 3-month.

^d^AtBM: Attentional Bias Modification; ApBM: Approach Bias Modification.

^e^For QSU, BDI, and MTQ: at each assessment time-point, average score at that assessment time-point is reported; at each Time/Time Phase, time average score at that time phase is reported.

**Table S2.** Results of Multilevel Modeling (MLM) analyses for additional secondary outcomes

| Omnibus effects^b^ | QSU^c^ | BDI | MTQ |
| --- | --- | --- | --- |
| Time/Time Phase | *F*(3, 344.37) = 19.39,  ***P* < .001^a^** | *F*(3, 340.94) = 4.06,  ***P* = .007** | *F*(2, 1244.43) = 0.09,  *P* = .92 |
| AtBM | *F*(1, 749.63) = 0.21,  *P* = .65 | *F*(1, 772.67) = 0.19,  *P* = .66 | *F*(1, 413.94) = 0.01,  *P* = .90 |
| ApBM | *F*(1, 749.63) = 0.68,  *P* = .41 | *F*(1, 772.67) = 6.31,  *P* = .01 | *F*(1, 413.94) = 6.81,  *P* = .01 |
| Time/Time Phase × AtBM | *F*(3, 344.37) = 3.18,  *P* = .02 | *F*(3, 340.94) = 0.63,  *P* = .59 | *F*(2, 1244.43) = 0.15,  *P* = .86 |
| Time/Time Phase × ApBM | *F*(3, 344.37) = 0.27,  *P* = .84 | *F*(3, 340.94) = 1.72,  *P* = .16 | *F*(2, 1244.43) = 5.10,  ***P* = .006** |
| AtBM × ApBM | *F*(1, 749.63) = 0.02,  *P* = .89 | *F*(1, 772.67) = 0.80,  *P* = .37 | *F*(1, 413.94) = 0.001,  *P* = .97 |
| Time/Time Phase × AtBM × ApBM | *F*(3, 344.37) = 2.78,  *P* = .04 | *F*(3, 340.94) = 2.32,  *P* = .08 | *F*(2, 1244.43) = 0.05,  *P* = .96 |

^a^Bold print: significant effects maintained after Holm-Bonferroni correction.

^b^AtBM: Attentional Bias Modification; ApBM: Approach Bias Modification.

^c^QSU: Questionnaire on Smoking Urges. BDI: Beck Depression Inventory Fast Screen. MTQ: Motivation to quit

smoking.

**Table S3.** Full MLM model for craving (QSU)

| **Fixed effects^b^** | ***B (95%CI)^a^*** | ***t value (df)*** | ***P value*** |
| --- | --- | --- | --- |
| Intercept | 31.40 (28.85, 33.95) | 24.03 (565.91) | < .001 |
| Time Phase (TP2) | -6.38 (-11.75, -1.02) | -2.32 (406.55) | .02 |
| Time Phase (TP3) | -7.35 (-13.41, -1.29) | -2.37 (390.62) | .02 |
| Time Phase (TP4) | -2.53 (-7.33, 2.28) | -1.03 (497.30) | .31 |
| AtBM | 4.19 (0.60, 7.79) | 2.28 (565.91) | .02 |
| ApBM | 2.79 (-0.77, 6.35) | 1.53 (565.91) | .13 |
| TP2 × AtBM | -1.48 (-8.75, 5.80) | -0.40 (403.43) | .69 |
| TP3 × AtBM | -5.46 (-14.45, 3.53) | -1.18 (379.58) | .24 |
| TP4 × AtBM | -14.20 (-21.28, -7.12) | -3.91 (479.48) | < .001 |
| TP2 × ApBM | 1.45 (-5.65, 8.54) | 0.40 (402.05) | .69 |
| TP3 × ApBM | -3.34 (-12.71, 6.03) | -0.70 (370.65) | .49 |
| TP4 × ApBM | -4.25 (-11.48, 2.98) | -1.15 (471.36) | .25 |
| AtBM × ApBM | -4.54 (-9.75, 0.66) | -1.70 (565.91) | .09 |
| TP2 × AtBM × ApBM | -1.29 (-11.40, 8.82) | -0.25 (399.70) | .80 |
| TP3 × AtBM × ApBM | 8.39 (-4.69, 21.46) | 1.25 (372.27) | .21 |
| TP4 × AtBM × ApBM | 13.17 (2.99, 23.35) | 2.52 (470.52) | .01 |

^a^95%CI: 95% confidence interval.

^b^AtBM: Attentional Bias Modification. ApBM: Approach Bias Modification. TP2: first half

intervention phases. TP3: second half intervention phase. TP4: follow-up phase. For Time Phase:

TP1 (baseline) was the reference category; for AtBM or ApBM, sham version of the training was

the reference category.

**Table S4.** Full MLM model for depression severity (BDI)

| **Fixed effects^b^** | ***B (95%CI)^a^*** | ***t value (df)*** | ***P value*** |
| --- | --- | --- | --- |
| Intercept | 3.79 (3.20, 4.38) | 12.55 (561.53) | < .001 |
| Time Phase (TP2) | 0.62 (-0.52, 1.75) | 1.06 (387.87) | .29 |
| Time Phase (TP3) | 0.60 (-0.68, 1.88) | 0.91 (375.42) | .36 |
| Time Phase (TP4) | 0.21 (-0.82, 1.23) | 0.39 (444.85) | .70 |
| AtBM | 0.41 (-0.42, 1.24) | 0.97 (561.53) | .33 |
| ApBM | 0.18 (-0.64, 1.00) | 0.42 (561.53) | .67 |
| TP2 × AtBM | -1.05 (-2.59, 0.49) | -1.33 (385.71) | .18 |
| TP3 × AtBM | -1.66(-3.55,0.24) | -1.71 (366.85) | .09 |
| TP4 × AtBM | -1.15 (-2.66, 0.35) | -1.49 (431.86) | .14 |
| TP2 × ApBM | -1.60 (-3.10, -0.10) | -2.08 (384.76) | .04 |
| TP3 × ApBM | -2.16 (-4.13, -0.19) | -2.14 (360.14) | .03 |
| TP4 × ApBM | -2.60 (-4.14, -1.06) | -3.29 (425.83) | .001 |
| AtBM × ApBM | -0.92 (-2.12, 0.29) | -1.48 (561.53) | .14 |
| TP2 × AtBM × ApBM | 1.46 (-0.68, 3.60) | 1.33 (383.13) | .18 |
| TP3 × AtBM × ApBM | 2.30 (-0.45, 5.06) | 1.63 (361.38) | .10 |
| TP4 × AtBM × ApBM | 2.87 (0.70, 5.04) | 2.58 (425.48) | .01 |

^a^95%CI: 95% confidence interval.

^b^AtBM: Attentional Bias Modification. ApBM: Approach Bias Modification. TP2: first half

intervention phases. TP3: second half intervention phase. TP4: follow-up phase. For Time Phase:

TP1 (baseline) was the reference category; for AtBM or ApBM, sham version of the training was

the reference category.

**Table S5.** Full MLM model for motivation to quit (MTQ)

| **Fixed effects^b^** | ***B (95%CI)^a^*** | ***t value (df)*** | ***P value*** |
| --- | --- | --- | --- |
| Intercept | 65.88 (60.64, 71.12) | 24.52 (622.61) | < .001 |
| Time Phase (TP2) | 2.02 (-2.72, 6.76) | 0.83 (1309.18) | .41 |
| Time Phase (TP3) | -2.31 (-8.40, 3.78) | -0.74 (1302.80) | .46 |
| AtBM | 0.52 (-6.61, 7.64) | 0.14 (662.61) | .89 |
| ApBM | 4.80 (-2.36, 11.95) | 1.31 (662.61) | .19 |
| TP2 × AtBM | -1.92 (-8.52, 4.69) | -0.57 (1319.44) | .57 |
| TP3 × AtBM | -0.74 (-9.43, 7.95) | -0.17 (1307.20) | .87 |
| TP2 × ApBM | -2.23 (-8.66, 4.21) | -0.67 (1303.50) | .50 |
| TP3 × ApBM | 6.46 (-2.12, 15.03) | 1.47 (1291.17) | .14 |
| AtBM × ApBM | -0.46 (-10.79, 9.87) | -0.09 (662.61) | .93 |
| TP2 × AtBM × ApBM | 1.36 (-8.15, 10.86) | 0.28 (1319.26) | .78 |
| TP3 × AtBM × ApBM | 0.48 (-11.97, 12.92) | 0.07 (1305.55) | .94 |

^a^95%CI: 95% confidence interval.

^b^AtBM: Attentional Bias Modification. ApBM: Approach Bias Modification. TP2: first half

intervention phases. TP3: second half intervention phase. For Time Phase: TP1 (baseline) was

the reference category; for AtBM or ApBM, sham version of the training was the reference category.

The results of the MLM analysis showed only a significant main effect of Time on craving (see Table S2). Participants in all conditions showed a significant reduction in craving from baseline to the first half of the intervention (*B* = -6.38, *95% CI* = [-11.75, -1.02], *P* = .02, *d* = 0.42); this reduction was maintained in the second half of the intervention (*B* = -7.35, *95% CI* = [-13.41, -1.29], *P* = .02, *d* = 0.48) but was not maintained in the follow-up phase (*B* = -2.53, *95%CI* = [-7.33, 2.28], *P* = .31, *d* = 0.17). Although there was also a main effect of Time on depression symptoms (see Table S2), follow-up analyses showed none of the regression coefficients involving Time were significant (see Table S4). There were no interaction effects between training version and Time for all measures, except for a significant interaction effect of ApBM × Time Phase on motivation to quit smoking (see Table S2). However, in a follow-up analysis, the effect could not be attributed to any of the training conditions as none of the regression coefficients for the interaction Time Phases × ApBM were significant (see Table S5).

## References

1. Kushnir V, Menon M, Balducci XL, Selby P, Busto U, Zawertailo L. Enhanced smoking cue salience associated with depression severity in nicotine-dependent individuals: a preliminary fMRI study. Int J Neuropsychopharmacol 2013 Jun;16(5):997-1008. [doi: 10.1017/S1461145710000696] PMID: 20604987

2. Vangeli E, Stapleton J, Smit ES, Borland R, West R. Predictors of attempts to stop smoking and their success in adult general population samples: A systematic review. Addiction 2011 Dec;106(12):2110-2121. [doi: 10.1111/j.1360-0443.2011.03565.x] PMID: 21752135

3. Field M, Munafò MR, Franken IH. A meta-analytic investigation of the relationship between attentional bias and subjective craving in substance abuse. Psychol Bull 2009 Jul;135(4):589-607. [doi: 10.1037/a0015843] PMID: 19586163

4. West R, Ussher M. Is the ten-item Questionnaire of Smoking Urges (QSU-brief) more sensitive to abstinence than shorter craving measures? Psychopharmacology (Berl) 2010 Feb;208(3):427–432. [doi: 10.1007/s00213-009-1742-x] PMID: 20033133

5. Poole H, Bramwell R, Murphy P. The utility of the Beck Depression Inventory Fast Screen (BDI-FS) in a pain clinic population. Eur J Pain 2009 Sep;13(8):865–869. [doi: 10.1016/j.ejpain.2008.09.017] PMID:19010075
