# Supplementary material for: Combining Web-Based Attentional Bias Modification and Approach Bias Modification as a Self-Help Smoking Intervention for Adult Smokers Seeking Online Help: Double-Blind Randomized Controlled Trial
Source: JMIR Ment Health. 2020 May 8;7(5):e16342. doi: 10.2196/16342 (PMC7244992; doi:10.2196/16342)
Supplement: Multimedia Appendix 2 [file mental_v7i5e16342_app2.doc]

## Multimedia Appendix 2. Exploratory moderation analyses

We explored whether participants were aware of the CBM condition they were assigned to and whether this moderated training effects on smoking behaviors.

### Methods

At the end of the training evaluation, participants’ awareness of which training version they completed was assessed for each training type: “There are two versions of the Attention training (i.e., AtBM) [or Action training (i.e., ApBM)]: a version we expect much effect and a version we expect little or no effect. Which version do you think you had (the version we expect much effect (i.e., the active version) or the version we expect little or no effect (i.e., the sham version))?”

The potential moderating effect of awareness of training version was analyzed separately for the AtBM and ApBM training. To test if awareness of training version would moderate the training effects on smoking behaviors, we tested including AtBM (active vs. sham), Time Phase (TP2, TP3, TP4 vs. TP1), awareness of assigned AtBM condition (correct vs. incorrect), and their interaction; or ApBM (active vs. sham), Time Phase (TP2, TP3, TP4 vs. TP1), awareness of assigned ApBM (correct vs. incorrect), and their interaction. The effects of interest in the moderation analyses were the three-way interactions between AtBM, Time Phase, and awareness of AtBM condition, or between ApBM, Time Phase, and awareness of ApBM condition. When evidence of a three-way interaction effect was present, the three-way interaction was decomposed to a two-way interaction between Time Phase and awareness of training condition, which was tested separately for participants in the active training condition and in the sham training condition. Holm-Bonferroni correction was applied to all analyses to account for multiple testing.

### Results

In total, 19.6% (99/504) of the final sample (i.e., TEQ respondents) indicated their awareness of training condition. Among the participants, 83% (43/52) and 70% (38/54) correctly realized that they were in the sham-training condition for AtBM and ApBM, respectively; however, only 34% (16/47) and 33% (15/45) correctly realized that they were in the active-training condition for AtBM and ApBM, respectively (*χ*^2^(1) = 24.3, *P* < .001; *χ*^2^(1) = 13.5, *P* < .001). In sum, although the large majority of the participants in the sham-training conditions were aware of this fact, in the active-training conditions, a (smaller) majority also thought they were in a sham-training condition.

Moderation analyses were conducted in this sub-sample (n = 99). MLM analysis indicated that there were interaction effects of AtBM × Time Phase × awareness of AtBM (*F*(3, 923.69) = 18.61, *P* < .001), and of ApBM × Time Phase × awareness of ApBM (*F*(3, 924.05) = 9.66, *P* < .001) on daily cigarette use, suggesting that awareness of CBM condition moderated the training effects in daily cigarette use. Specifically, participants who correctly thought that they were in the active training condition showed larger decreases in daily cigarette use from baseline to the second half of the intervention (for AtBM: *B* = -7.87, *95% CI* = [-11.59, -4.14], *P* < .001, *d* = 0.71; for ApBM: *B* = -4.39, *95% CI* = [-8.26, -0.52], *P* = 0.03, *d* = 0.40) and to the follow-up phase (for AtBM: *B* = -9.14, *95% CI* = [-13.13, -5.16], *P* < .001, *d* = 0.83; for ApBM: *B* = -5.33, *95% CI* = [-9.46, -1.19], *P* = 0.01, *d* = 0.48), compared to those who thought they were in the sham training condition but actually they received the active training condition. This difference did not appear in the sham training condition for both AtBM and ApBM (the effects did not remain after Holm-Bonferroni correction; see Figure S1 and S2).


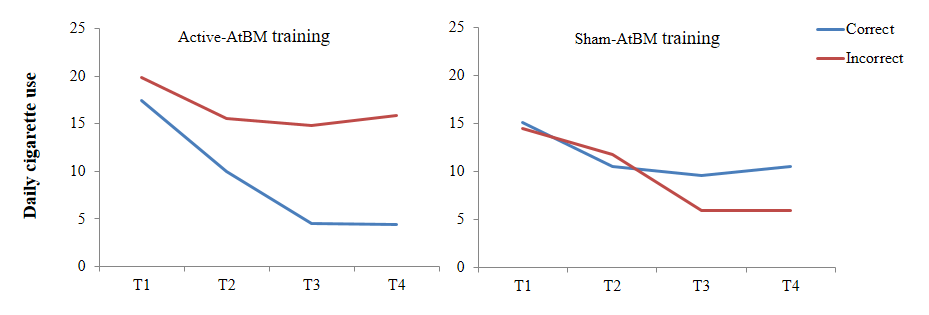


**Figure S1.** Interaction between AtBM, Time Phase, and awareness of AtBM condition in predicting daily cigarette use. Correct: participants correctly realized their training condition. Incorrect: participants incorrectly realized their training condition. For each intervention phase: T1 (baseline), T2 (first half of the intervention), T3 (second half of the intervention), and T4 (follow-up). n = 16 and 31 participants correctly and incorrectly realized they were in the active AtBM training condition, respectively; n = 43 and 9 participants correctly and incorrectly realized they were in the sham AtBM training condition, respectively.


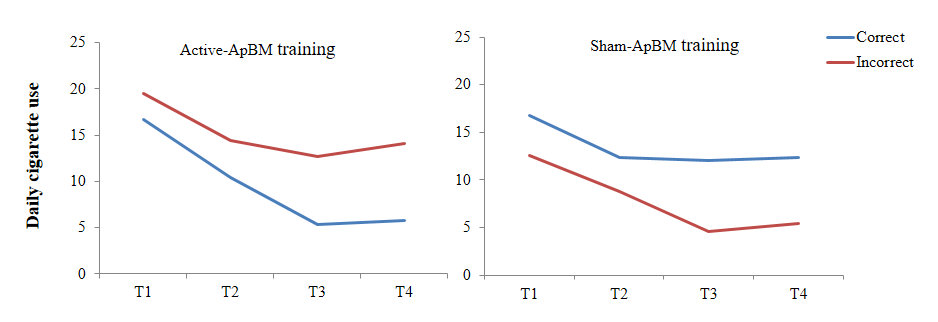


**Figure S2.** Interaction between ApBM, Time, and awareness of ApBM condition in predicting daily cigarette use. Correct: participants correctly realized their training condition. Incorrect: participants incorrectly realized their training condition. For each intervention phase: T1 (baseline), T2 (first half of the intervention), T3 (second half of the intervention), and T4 (follow-up). n = 15 and 30 participants correctly and incorrectly realized they were in the active ApBM training condition, respectively; n = 38 and 16 participants correctly and incorrectly realized they were in the sham ApBM training condition, respectively.
