# Supplementary material for: Combining Web-Based Attentional Bias Modification and Approach Bias Modification as a Self-Help Smoking Intervention for Adult Smokers Seeking Online Help: Double-Blind Randomized Controlled Trial
Source: JMIR Ment Health. 2020 May 8;7(5):e16342. doi: 10.2196/16342 (PMC7244992; doi:10.2196/16342)
Supplement: Multimedia Appendix 3 [file mental_v7i5e16342_app3.doc]

## Multimedia Appendix 3. Task stimuli

Picture stimuli used for cognitive bias assessment and training were created following the same stimulus development protocol of the Amsterdam Beverage Picture Set [1], by creating 180 smoking-related and 180 matched neutral pictures in both passive (i.e., a package of cigarettes or a box of pencils) and active (i.e., a person lighting up a cigarette or holding a pencil) contexts.

The four main assessments and the 11 mini-assessment blocks at the start of each training session included both untrained and trained (i.e., presented during the previous training sessions) pictures, except for the first assessment and training session. The baseline, mid- and post-training assessments included 10 untrained picture pairs (i.e., 10 smoking-related and 10 matched neutral pictures) and ten trained picture pairs presented in the previous training session for each task (except for the baseline assessment, which presented 20 untrained picture pairs). Similarly, the mini-assessment blocks at the start of each training task included four untrained picture pairs and four pairs trained in the previous training session (except for the first session, which presented eight untrained picture pairs). The 3-month follow-up assessment presented the same pictures used in the post-training assessment.

Each training session presented 12 picture pairs randomly picked out of the remaining stimuli not used for the assessment sessions. In order to ensure a sufficiently large number of pictures for training, from training session six onwards the four untrained picture pairs presented in the mini-assessment blocks of training sessions one to five were re-entered in the pool of pictures used for training. An automatic counting system kept track of the amount of times the pictures were used for training over the two tasks, in order to balance picture repetition. All sets of pictures presented for assessment and training were always stratified by context (i.e., active and passive).

The task stimuli can be requested from the first author.

## References

1. Pronk T, van Deursen DS, Beraha EM, Larsen H, Wiers RW. Validation of the Amsterdam Beverage Picture Set: A Controlled Picture Set for Cognitive Bias Measurement and Modification Paradigms. Alcohol Clin Exp Res 2015 Oct; 39(10):2047–2055. [doi: 10.1111/acer.12853] PMID: 26431117
