# Supplementary material for: Combining Web-Based Attentional Bias Modification and Approach Bias Modification as a Self-Help Smoking Intervention for Adult Smokers Seeking Online Help: Double-Blind Randomized Controlled Trial
Source: JMIR Ment Health. 2020 May 8;7(5):e16342. doi: 10.2196/16342 (PMC7244992; doi:10.2196/16342)
Supplement: Multimedia Appendix 4 [file mental_v7i5e16342_app4.doc]

## Multimedia Appendix 4. Task data preparation

Before computing the AtB and ApB indexes, the following task trials were excluded: 1) practice trials, 2) incorrect trials (ranging from 3.68% to 7.07% for the VPT, and from 3.24% to 5.77% for the AAT), and 3) correct repetitions of incorrect trials [1,2]. Additionally, for both the VPT and AAT, data of participants with an error rate greater than 35% were excluded (VPT: n = 1 at baseline and n = 1 at mid-training assessment; AAT: n = 4 at baseline, n = 1 at interim assessment 2, and n = 1 at interim assessment 4; [3]). Moreover, one extreme outlier was found (> 8SDs from the group’s mean) in the baseline AtB score distribution and was excluded from the analysis.

For the VPT task, it is assumed that discrimination of the probe direction will be quicker when probes appear in the locus participants are already attending to, that is, in the case of smokers, on the smoking-related stimuli. Therefore, it is usually expected that participants would respond quicker when the probe appeared at the location of the smoking-related pictures than when it appeared at the location of the neutral pictures (i.e., AtB for smoking stimuli). Additionally, we also expected longer reaction times (slow responses) on “top” trials, particularly when the probe appeared on top of the neutral pictures than when it replaced them, since it would be more difficult for participants to disengage from the smoking-related pictures, which stayed on the computer screen. To test these hypotheses, a within-subjects ANOVA was performed to compare the VPT mean reaction times for “top” trials against “after” trials for the two types of pictures at baseline. The results showed that there was a main effect of picture type (*F*(1, 494) = 346.68, *P* < 0.001; RTs for smoking trials: *M* = 649.88, *SD* = 5.12; RTs for neutral trials: *M* = 676.07, *SD* = 5.41) and trial type (*F*(1, 494) = 166.77, *p* < 0.001; RTs for “top” trials: *M* = 652.77, *SD* = 5.39; RTs for “after” trials: *M* = 673.18, *SD* = 5.17). However, there was no interaction effect between trial type and picture type (*F*(1, 494) = 0.10, *P* = 0.75). These results suggested that there was no indication of a difference in AtB scores between “after” and “top” trials in the whole sample. Thus, we combined the two AtB scores (i.e., AtB score for “top” and “after” trials) into a single AtB index.

## References

1. Elfeddali I, de Vries H, Bolman C, Pronk T, Wiers RW. A randomized controlled trial of Web-based Attentional Bias Modification to help smokers quit. Heal Psychol 2016 Aug;35(8):870-880. [doi: 10.1037/hea0000346] PMID: 27505210

2. Kong G, Larsen H, Cavallo DA, Becker D, Cousijn J, Salemink E, Collot D’Escury-Koenigs AL, Morean ME, Wiers RW, Krishnan-Sarin S. Re-training automatic action tendencies to approach cigarettes among adolescent smokers: A pilot study. Am J Drug Alcohol Abuse 2015;41(5):425-432. [doi: 10.3109/00952990.2015.1049492] PMID: 26186485

3. Wittekind CE, Reibert E, Takano K, Ehring T, Pogarell O, Rüther T. Approach-avoidance modification as an add-on in smoking cessation: A randomized controlled study. Behav Res Ther 2019 Mar;114:35-43. [doi: 10.1016/j.brat.2018.12.004] PMID: 30716613
