# Supplementary material for: Combining Web-Based Attentional Bias Modification and Approach Bias Modification as a Self-Help Smoking Intervention for Adult Smokers Seeking Online Help: Double-Blind Randomized Controlled Trial
Source: JMIR Ment Health. 2020 May 8;7(5):e16342. doi: 10.2196/16342 (PMC7244992; doi:10.2196/16342)
Supplement: Multimedia Appendix 5 [file mental_v7i5e16342_app5.doc]

## Multimedia Appendix 5. Summary of training evaluation responses in the training condition of AtBM and ApBM, respectively.

**Table S6.** Summary of training evaluation responses in the training condition of AtBM

|  | active-AtBM | sham-AtBM | *F*(1, 97)  /*χ*^2^(1) | *P value* |
| --- | --- | --- | --- | --- |
|  | (n = 47) | (n = 52) |
| ***Training evaluation for CBM^a^ as a whole*** |  |  |  |  |
| 1. What do you think of the quality of  this CBM training?^b^, n (%) | 23 (48.9) | 24 (46.2) | 0.08 | .78 |
| 2. How satisfied are you overall with  this CBM training?^c^, n (%) | 24 (51.1) | 23 (44.2) | 0.46 | .50 |
| 3. I think the CBM training helped me with  my problems^d^, *M* (*SD*) | 3.32 (1.95) | 3.54 (2.03) | 0.30 | .59 |
| 4. Would you recommend this CBM training  to others?^e^, n (%) | 26 (55.3) | 22 (42.3) | 1.67 | .20 |
| 5. Will you use the CBM training in the further?^e^, n (%) | 32 (68.1) | 33 (63.5) | 0.23 | .63 |
| ***Training evaluation for AtBM^a^*** |  |  |  |  |
| 1. The goal of the AtBM training was clear  before I started it^d^, *M* (*SD*) | 5.06 (1.79) | 4.90 (1.90) | 0.19 | .67 |
| 2. The instructions on what I should do during the  AtBM training was clear^d^, *M* (*SD*) | 5.98 (1.66) | 6.12 (1.47) | 0.19 | .66 |
| 3. The AtBM training was difficult to do^d^ *M* (*SD*) | 3.30 (1.90) | 3.21 (1.79) | 0.05 | .82 |
| 4. The AtBM training was fun to do^d^, *M* (*SD)* | 3.32 (1.91) | 3.27 (1.96) | 0.02 | .90 |
| ***Training evaluation for ApBM^a^*** |  |  |  |  |
| 1. The goal of the ApBM training was clear  before I started it^d^, *M* (*SD)* | 5.28 (1.69) | 4.96 (2.04) | 0.69 | .41 |
| 2. The instructions on what I should do during the  ApBM training was clear^d^, *M* (*SD)* | 6.11 (1.46) | 5.87 (1.50) | 0.65 | .42 |
| 3. The ApBM training was difficult to do^d^, *M* (*SD*) | 2.81 (1.78) | 2.96 (1.83) | 0.18 | .67 |
| 4. The ApBM training was fun to do^d^, *M* (*SD*) | 3.72 (2.03) | 3.88 (2.12) | 0.15 | .70 |

^a^CBM: Cognitive Bias Modification; AtBM: Attentional Bias Modification; ApBM: Approach Bias Modification.

^b^Poor, fair, good, excellent; percentage of “good” and “excellent” responses.

^c^Very dissatisfied, fairly dissatisfied, fairly satisfied, very satisfied; percentage of “fairly satisfied” and “very satisfied”

responses.

^d^Participants indicated the extent to which they agreed with this statement on a scale from 1 (completely disagree) to 7

(completely agree)

^e^No, definitely not, No, I do not think so, Yes, I think so, Yes, definitely; percentage of “Yes, I think so” and “Yes,

definitely” responses.

**Table S7.** Summary of training evaluation responses in the training condition of ApBM

|  | active-ApBM | sham-ApBM | *F*(1, 97)  /*χ*^2^(1) | *P value* |
| --- | --- | --- | --- | --- |
|  | (n = 45) | (n = 54) |
| ***Training evaluation for CBM^a^ as a whole*** |  |  |  |  |
| 1. What do you think of the quality of  this CBM training?^b^, n (%) | 21 (46.7) | 26 (48.1) | 0.02 | .88 |
| 2. How satisfied are you overall with  this CBM training?^c^, n (%) | 22 (48.9) | 25 (46.3) | 0.07 | .80 |
| 3. I think the CBM training helped me with  my problems^d^, *M* (*SD*) | 3.36 (2.12) | 3.50 (1.88) | 0.13 | .72 |
| 4. Would you recommend this CBM training  to others?^e^, n (%) | 22 (48.9) | 26 (48.1) | 0.01 | .94 |
| 5. Will you use the CBM training in the further?^e^, n (%) | 30 (66.7) | 35 (64.8) | 0.04 | .85 |
| ***Training evaluation for AtBM^a^*** |  |  |  |  |
| 1. The goal of the AtBM training was clear  before I started it^d^, *M* (*SD*) | 5.22 (1.61) | 4.78 (2.01) | 1.44 | .23 |
| 2. The instructions on what I should do during the  AtBM training was clear^d^, *M* (*SD*) | 6.22 (1.35) | 5.91 (1.71) | 1.01 | .32 |
| 3. The AtBM training was difficult to do^d^ *M* (*SD*) | 3.11 (1.84) | 3.37 (1.84) | 0.49 | .49 |
| 4. The AtBM training was fun to do^d^, *M* (*SD)* | 3.27 (1.88) | 3.31 (1.99) | 0.02 | .90 |
| ***Training evaluation for ApBM^a^*** |  |  |  |  |
| 1. The goal of the ApBM training was clear  before I started it^d^, *M* (*SD)* | 5.29 (1.84) | 4.96 (1.91) | 0.74 | .39 |
| 2. The instructions on what I should do during the  ApBM training was clear^d^, *M* (*SD)* | 6.02 (1.32) | 5.94 (1.61) | 0.07 | .80 |
| 3. The ApBM training was difficult to do^d^, *M* (*SD*) | 2.60 (1.64) | 3.13 (1.89) | 2.16 | .15 |
| 4. The ApBM training was fun to do^d^, *M* (*SD*) | 3.98 (2.13) | 3.67 (2.03) | 0.55 | .46 |

^a^CBM: Cognitive Bias Modification; AtBM: Attentional Bias Modification; ApBM: Approach Bias Modification.

^b^Poor, fair, good, excellent; percentage of “good” and “excellent” responses.

^c^Very dissatisfied, fairly dissatisfied, fairly satisfied, very satisfied; percentage of “fairly satisfied” and “very satisfied”

responses.

^d^Participants indicated the extent to which they agreed with this statement on a scale from 1 (completely disagree) to 7

(completely agree)

^e^No, definitely not, No, I do not think so, Yes, I think so, Yes, definitely; percentage of “Yes, I think so” and “Yes,

definitely” responses.
