# Supplementary material for: Combining Web-Based Attentional Bias Modification and Approach Bias Modification as a Self-Help Smoking Intervention for Adult Smokers Seeking Online Help: Double-Blind Randomized Controlled Trial
Source: JMIR Ment Health. 2020 May 8;7(5):e16342. doi: 10.2196/16342 (PMC7244992; doi:10.2196/16342)
Supplement: Multimedia Appendix 6 [file mental_v7i5e16342_app6.doc]

## Multimedia Appendix 6. Full MLM models for primary and secondary outcomes

**Table S8.** Full MLM model for point prevalence abstinence (PPA)

| **Fixed effects^b^** | ***B*** | ***OR (95%CI)^a^*** | ***Z score*** | ***P value*** |
| --- | --- | --- | --- | --- |
| Intercept | -6.66 | 0.00 (0.00, 0.01) | -8.79 | < .001 |
| Time Phase (TP2) | 1.12 | 3.07 (0.97, 9.74) | 1.90 | .06 |
| Time Phase (TP3) | 0.45 | 1.57 (0.38, 6.39) | 0.63 | .53 |
| AtBM | -0.09 | 0.91 (0.21, 3.95) | -0.12 | .90 |
| ApBM | -0.53 | 0.59 (0.13, 2.62) | -0.70 | .49 |
| TP2 × AtBM | 0.04 | 1.04 (0.15, 7.28) | 0.04 | .97 |
| TP3 × AtBM | 2.44 | 11.52 (0.68, 196.41) | 1.69 | .09 |
| TP2 × ApBM | -0.19 | 0.83 (0.17, 4.04) | -0.23 | .82 |
| TP3 × ApBM | 0.96 | 2.60 (0.33, 20.49) | 0.91 | .36 |
| AtBM × ApBM | 0.74 | 2.09 (0.24, 17.93) | 0.67 | .50 |
| TP2 × AtBM × ApBM | 1.67 | 5.31 (0.42, 67.04) | 1.29 | .20 |
| TP3 × AtBM × ApBM | -1.61 | 0.20 (0.01, 6.87) | -0.89 | .37 |

^a^OR: Odds Ratios; 95%CI: 95% confidence interval.

^b^AtBM: Attentional Bias Modification. ApBM: Approach Bias Modification. TP2: second half intervention phase. TP3: follow-up phase. For Time Phase: TP1 (first half intervention phases) was the reference category; for AtBM or ApBM, sham version of the training was the reference category.

**Table S9.** Full MLM model for daily cigarette use (DCU)

| **Fixed effects^b^** | ***B (95%CI)^a^*** | ***t value (df)*** | ***P value*** |
| --- | --- | --- | --- |
| Intercept | 15.20 (13.65, 16.75) | 19.10 (699.90) | < .001 |
| Time Phase (TP2) | -3.89 (-4.96, -2.83) | -7.13 (1933) | < .001 |
| Time Phase (TP3) | -5.46 (-6.91, -4.00) | -7.31 (1911) | < .001 |
| Time Phase (TP4) | -4.61 (-6.32, -2.90) | -5.25 (1875) | < .001 |
| AtBM | 1.51 (-0.68, 3.69) | 1.34 (699.90) | .18 |
| ApBM | 0.22 (-1.94, 2.39) | 0.20 (699.90) | .84 |
| TP2 × AtBM | -0.72 (-2.19, 0.75) | -0.96 (1905) | .34 |
| TP3 × AtBM | -0.26 (-2.32, 1.80) | -0.25 (1892) | .80 |
| TP4 × AtBM | -1.38 (-3.90, 1.14) | -1.07 (1857) | .28 |
| TP2 × ApBM | -0.002 (-1.46, 1.45) | -0.003 (1916) | .99 |
| TP3 × ApBM | -0.24 (-2.27, 1.78) | -0.23 (1896) | .81 |
| TP4 × ApBM | -1.52 (-4.07, 1.04) | -1.16 (1855) | .25 |
| AtBM × ApBM | -1.68 (-4.85, 1.49) | -1.03 (699.90) | .30 |
| TP2 × AtBM × ApBM | 0.005 (-2.12, 2.13) | 0.004 (1908) | .99 |
| TP3 × AtBM × ApBM | -2.48 (-5.42, 0.46) | -1.65 (1894) | .10 |
| TP4 × AtBM × ApBM | 0.61 (-3.02, 4.25) | 0.33 (1856) | .74 |

^a^95%CI: 95% confidence interval.

^b^AtBM: Attentional Bias Modification. ApBM: Approach Bias Modification. TP2: first half

intervention phases. TP3: second half intervention phase. TP4: follow-up phase. For Time Phase:

TP1 (baseline) was the reference category; for AtBM or ApBM, sham version of the training was

the reference category.

**Table S10.** Full MLM model for attentional bias (AtB)

| **Fixed effects^b^** | ***B (95%CI)^a^*** | ***t value (df)*** | ***P value*** |
| --- | --- | --- | --- |
| Intercept | 24.64 (18.94, 30.35) | 8.42 (1614.75) | < .001 |
| Time Phase (TP2) | -4.64 (-11.19, 1.92) | -1.38 (2016.39) | .17 |
| Time Phase (TP3) | -10.28 (-18.98, -1.58) | -2.30 (2036.09) | .02 |
| Time Phase (TP4) | -11.03 (-24.25, 1.99) | -1.65 (1943.93) | .10 |
| AtBM | 2.74 (-5.33, 10.80) | 0.66 (1618.13) | .51 |
| ApBM | 0.13 (-7.86, 8.12) | 0.03 (1619.84) | .97 |
| TP2 × AtBM | -12.19 (-21.43, -2.94) | -2.57 (2006.82) | .01 |
| TP3 × AtBM | -10.14 (-22.58, 2.30) | -1.59 (2038.66) | .11 |
| TP4 × AtBM | -15.73 (-35.40, 3.95) | -1.56 (1927.23) | .12 |
| TP2 × ApBM | -1.24 (-10.29, 7.80) | -0.27 (2000.23) | .79 |
| TP3 × ApBM | -7.66 (-19.96, 4.64) | -1.22 (2041.28) | .22 |
| TP4 × ApBM | -13.80 (-33.94, 6.33) | -1.34 (1911.36) | .18 |
| AtBM × ApBM | -3.06 (-14.79, 8.67) | -0.51 (1620.96) | .61 |
| TP2 × AtBM × ApBM | 8.51 (-4.86, 21.89) | 1.24 (2005.95) | .21 |
| TP3 × AtBM × ApBM | 17.25 (-0.57, 35.08) | 1.89 (2040.15) | .06 |
| TP4 × AtBM × ApBM | 28.37 (-0.23, 56.96) | 1.93 (1920.88) | .053 |

^a^95%CI: 95% confidence interval.

^b^AtBM: Attentional Bias Modification. ApBM: Approach Bias Modification. TP2: first half

intervention phases. TP3: second half intervention phase. TP4: follow-up phase. For Time Phase:

TP1 (baseline) was the reference category; for AtBM or ApBM, sham version of the training was

the reference category.

**Table S11.** Full MLM model for approach bias (ApB)

| **Fixed effects^b^** | ***B (95%CI)^a^*** | ***t value (df)*** | ***P value*** |
| --- | --- | --- | --- |
| Intercept | 9.29 (-3.57, 22.14) | 1.41 (2049) | .16 |
| Time Phase (TP2) | -2.25 (-17.93, 13.43) | -0.28 (2049) | .78 |
| Time Phase (TP3) | -10.04 (-29.27, 9.20) | -1.02 (2049) | .31 |
| Time Phase (TP4) | 4.36 (-26.53, 35.25) | 0.28(2049) | .78 |
| AtBM | -0.47 (-18.64, 17.71) | -0.05 (2049) | .96 |
| ApBM | -4.77 (-22.77, 13.24) | -0.52 (2049) | .61 |
| TP2 × AtBM | -13.05 (-35.14, 9.05) | -1.15 (2049) | .25 |
| TP3 × AtBM | 12.21 (-15.35, 39.77) | 0.86 (2049) | .39 |
| TP4 × AtBM | -3.96 (-50.70, 42.79) | -0.17 (2049) | .87 |
| TP2 × ApBM | 1.45 (-20.26, 23.15) | 0.13 (2049) | .90 |
| TP3 × ApBM | 0.09 (-27.23, 27.42) | 0.007 (2049) | .99 |
| TP4 × ApBM | -16.69 (-64.61, 31.24) | -0.68 (2049) | .50 |
| AtBM × ApBM | -11.89 (-38.32, 14.53) | -0.88 (2049) | .38 |
| TP2 × AtBM × ApBM | 21.18 (-10.80, 53.16) | 1.29 (2049) | .20 |
| TP3 × AtBM × ApBM | -4.56 (-44.17, 35.05) | -0.23 (2049) | .82 |
| TP4 × AtBM × ApBM | 17.06 (-50.94, 85.06) | 0.49 (2049) | .63 |

^a^95%CI: 95% confidence interval.

^b^AtBM: Attentional Bias Modification. ApBM: Approach Bias Modification. TP2: first half

intervention phases. TP3: second half intervention phase. TP4: follow-up phase. For Time Phase:

TP1 (baseline) was the reference category; for AtBM or ApBM, sham version of the training was

the reference category.
