# Supplementary material for: Combining Web-Based Attentional Bias Modification and Approach Bias Modification as a Self-Help Smoking Intervention for Adult Smokers Seeking Online Help: Double-Blind Randomized Controlled Trial
Source: JMIR Ment Health. 2020 May 8;7(5):e16342. doi: 10.2196/16342 (PMC7244992; doi:10.2196/16342)
Supplement: Multimedia Appendix 7 [file mental_v7i5e16342_app7.pdf]

CONSORT-EHEALTH (V 1.6.1) - Submission/Publication Form

The CONSORT-EHEALTH checklist is intended for authors of randomized trials evaluating web-based and Internet-based applications/interventions, including mobile interventions, electronic games (incl multiplayer games), social media, certain telehealth applications, and other interactive and/or networked electronic applications. Some of the items (e.g. all subitems under item 5 - description of the intervention) may also be applicable for other study designs.

The goal of the CONSORT EHEALTH checklist and guideline is to be
a) a guide for reporting for authors of RCTs,
b) to form a basis for appraisal of an ehealth trial (in terms of validity)

CONSORT-EHEALTH items/subitems are MANDATORY reporting items for studies published in the Journal of Medical Internet Research and other journals / scientific societies endorsing the checklist.

Items numbered 1., 2., 3., 4a., 4b etc are original CONSORT or CONSORT-NPT (non-pharmacologic treatment) items.
Items with Roman numerals (i., ii, iii, iv etc.) are CONSORT-EHEALTH extensions/clarifications.

As the CONSORT-EHEALTH checklist is still considered in a formative stage, we would ask that you also RATE ON A SCALE OF 1-5 how important/useful you feel each item is FOR THE PURPOSE OF THE CHECKLIST and reporting guideline (optional).

Mandatory reporting items are marked with a red *.
In the textboxes, either copy & paste the relevant sections from your manuscript into this form - please include any quotes from your manuscript in QUOTATION MARKS,
or answer directly by providing additional information not in the manuscript, or elaborating on why the item was not relevant for this study.

YOUR ANSWERS WILL BE PUBLISHED AS A SUPPLEMENTARY FILE TO YOUR PUBLICATION IN JMIR AND ARE CONSIDERED PART OF YOUR PUBLICATION (IF ACCEPTED).
Please fill in these questions diligently. Information will not be copyedited, so please use proper spelling and grammar, use correct capitalization, and avoid abbreviations.

DO NOT FORGET TO SAVE AS PDF _AND_ CLICK THE SUBMIT BUTTON SO YOUR ANSWERS ARE IN OUR DATABASE !!!

Citation Suggestion (if you append the pdf as Appendix we suggest to cite this paper in the caption):
Eysenbach G, CONSORT-EHEALTH Group
CONSORT-EHEALTH: Improving and Standardizing Evaluation Reports of Web-based and Mobile Health Interventions
J Med Internet Res 2011;13(4):e126
URL: http://www.jmir.org/2011/4/e126/

doi: 10.2196/jmir.1923
PMID: 22209829

*Required

Your name *

First Last

Si Wen

Primary Affiliation (short), City, Country *

University of Toronto, Toronto, Canada

University of Amsterdam, Amsterdam, Netherl

Your e-mail address *

abc@gmail.com

si-wen@hotmail.com

Title of your manuscript *

Provide the (draft) title of your manuscript.

Combining Web-Based Attentional Bias Modification and Approach Bias Modification as a Self-Help Smoking Intervention for Adult Smokers Seeking Online Help: Double-Blind Randomized Controlled Trial

Name of your App/Software/Intervention *

If there is a short and a long/alternate name, write the short name first and add the long name in brackets.

S-CBM (Web-based Cognitive Bias Modificatio

Evaluated Version (if any)

e.g. "V1", "Release 2017-03-01", "Version 2.0.27913"

N/A.

Language(s) *

What language is the intervention/app in? If multiple languages are available, separate by comma (e.g. "English, French")

Dutch

URL of your Intervention Website or App

e.g. a direct link to the mobile app on app in appstore (itunes, Google Play), or URL of the website. If the intervention is a DVD or hardware, you can also link to an Amazon page.

http://www.impliciet.eu/trainings/roken/

URL of an image/screenshot (optional)

Your answer

Accessibility *

Can an enduser access the intervention presently?

- ○ access is free and open
- ○ access only for special usergroups, not open
- ○ access is open to everyone, but requires payment/subscription/in-app purchases
- ◉ app/intervention no longer accessible
- ○ Other:

Primary Medical Indication/Disease/Condition *

e.g. "Stress", "Diabetes", or define the target group in brackets after the condition, e.g. "Autism (Parents of children with)", "Alzheimers (Informal Caregivers of)"

Adult smokers seeking online help for smoking

Primary Outcomes measured in trial *

comma-separated list of primary outcomes reported in the trial

Point prevalence abstinence along the study p

Secondary/other outcomes

Are there any other outcomes the intervention is expected to affect?

Daily cigarette use, attentional bias, approach bias, craving, depression severity, and motivation to quit smoking

Recommended "Dose" *

What do the instructions for users say on how often the app should be used?

- ○ Approximately Daily
- ○ Approximately Weekly
- ○ Approximately Monthly
- ○ Approximately Yearly
- ○ "as needed"
- ◉ Other: Participants were suggested to complete the 11 training sessions within 4 wee

Approx. Percentage of Users (starters) still using the app as recommended after 3 months *

- (x) unknown / not evaluated
- ( ) 0-10%
- ( ) 11-20%
- ( ) 21-30%
- ( ) 31-40%
- ( ) 41-50%
- ( ) 51-60%
- ( ) 61-70%
- ( ) 71%-80%
- ( ) 81-90%
- ( ) 91-100%
- ( ) Other:

Overall, was the app/intervention effective? *

- ( ) yes: all primary outcomes were significantly better in intervention group vs control
- ( ) partly: SOME primary outcomes were significantly better in intervention group vs control
- ( ) no statistically significant difference between control and intervention
- ( ) potentially harmful: control was significantly better than intervention in one or more outcomes
- (x) inconclusive: more research is needed
- ( ) Other:

Article Preparation Status/Stage *

At which stage in your article preparation are you currently (at the time you fill in this form)

- ○ not submitted yet - in early draft status
- ○ not submitted yet - in late draft status, just before submission
- ○ submitted to a journal but not reviewed yet
- ○ submitted to a journal and after receiving initial reviewer comments
- ◉ submitted to a journal and accepted, but not published yet
- ○ published
- ○ Other:

Journal *

If you already know where you will submit this paper (or if it is already submitted), please provide the journal name (if it is not JMIR, provide the journal name under "other")

- ○ not submitted yet / unclear where I will submit this
- ○ Journal of Medical Internet Research (JMIR)
- ○ JMIR mHealth and UHealth
- ○ JMIR Serious Games
- ◉ JMIR Mental Health
- ○ JMIR Public Health
- ○ JMIR Formative Research
- ○ Other JMIR sister journal
- ○ Other:

Is this a full powered effectiveness trial or a pilot/feasibility trial? *

- (selected) Pilot/feasibility
- Fully powered

Manuscript tracking number *

If this is a JMIR submission, please provide the manuscript tracking number under "other" (The ms tracking number can be found in the submission acknowledgement email, or when you login as author in JMIR. If the paper is already published in JMIR, then the ms tracking number is the four-digit number at the end of the DOI, to be found at the bottom of each published article in JMIR)

- no ms number (yet) / not (yet) submitted to / published in JMIR
- (selected) Other: 16342

TITLE AND ABSTRACT

1a) TITLE: Identification as a randomized trial in the title

1a) Does your paper address CONSORT item 1a? *

I.e does the title contain the phrase "Randomized Controlled Trial"? (if not, explain the reason under "other")

- (selected) yes
- Other:

1a-i) Identify the mode of delivery in the title

Identify the mode of delivery. Preferably use “web-based” and/or “mobile” and/or “electronic game” in the title. Avoid ambiguous terms like “online”, “virtual”, “interactive”. Use “Internet-based” only if Intervention includes non-web-based Internet components (e.g. email), use “computer-based” or “electronic” only if offline products are used. Use “virtual” only in the context of “virtual reality” (3-D worlds). Use “online” only in the context of “online support groups”. Complement or substitute product names with broader terms for the class of products (such as “mobile” or “smart phone” instead of “iphone”), especially if the application runs on different platforms.

	1	2	3	4	5	
subitem not at all important	○	○	○	○	◉	essential

Does your paper address subitem 1a-i? *

Copy and paste relevant sections from manuscript title (include quotes in quotation marks "like this" to indicate direct quotes from your manuscript), or elaborate on this item by providing additional information not in the ms, or briefly explain why the item is not applicable/relevant for your study

"Web-Based Attentional Bias Modification and Approach Bias Modification"

1a-ii) Non-web-based components or important co-interventions in title

Mention non-web-based components or important co-interventions in title, if any (e.g., “with telephone support”).

	1	2	3	4	5	
subitem not at all important	○	○	◉	○	○	essential

Does your paper address subitem 1a-ii?

Copy and paste relevant sections from manuscript title (include quotes in quotation marks "like this" to indicate direct quotes from your manuscript), or elaborate on this item by providing additional information not in the ms, or briefly explain why the item is not applicable/relevant for your study

Your answer

1a-iii) Primary condition or target group in the title

Mention primary condition or target group in the title, if any (e.g., “for children with Type I Diabetes”)
Example: A Web-based and Mobile Intervention with Telephone Support for Children with Type I Diabetes: Randomized Controlled Trial

	1	2	3	4	5	
subitem not at all important	○	○	○	○	◉	essential

Does your paper address subitem 1a-iii? *

Copy and paste relevant sections from manuscript title (include quotes in quotation marks "like this" to indicate direct quotes from your manuscript), or elaborate on this item by providing additional information not in the ms, or briefly explain why the item is not applicable/relevant for your study

"for Adult Smokers Seeking Online Help"

1b) ABSTRACT: Structured summary of trial design, methods, results, and conclusions

NPT extension: Description of experimental treatment, comparator, care providers, centers, and blinding status.

1b-i) Key features/functionalities/components of the intervention and comparator in the METHODS section of the ABSTRACT

Mention key features/functionalities/components of the intervention and comparator in the abstract. If possible, also mention theories and principles used for designing the site. Keep in mind the needs of systematic reviewers and indexers by including important synonyms. (Note: Only report in the abstract what the main paper is reporting. If this information is missing from the main body of text, consider adding it)

	1	2	3	4	5	
subitem not at all important	○	○	○	○	◉	essential

Does your paper address subitem 1b-i? *

Copy and paste relevant sections from the manuscript abstract (include quotes in quotation marks "like this" to indicate direct quotes from your manuscript), or elaborate on this item by providing additional information not in the ms, or briefly explain why the item is not applicable/relevant for your study

"...in a double-blind randomized controlled trial (RCT) with a 2 (AtBM: active versus sham) × 2 (ApBM: active versus sham) factorial design"

1b-ii) Level of human involvement in the METHODS section of the ABSTRACT

Clarify the level of human involvement in the abstract, e.g., use phrases like “fully automated” vs. “therapist/nurse/care provider/physician-assisted” (mention number and expertise of providers involved, if any). (Note: Only report in the abstract what the main paper is reporting. If this information is missing from the main body of text, consider adding it)

	1	2	3	4	5	
subitem not at all important	○	○	○	◉	○	essential

Does your paper address subitem 1b-ii?

Copy and paste relevant sections from the manuscript abstract (include quotes in quotation marks "like this" to indicate direct quotes from your manuscript), or elaborate on this item by providing additional information not in the ms, or briefly explain why the item is not applicable/relevant for your study

"...to receive 11 fully automated CBM training sessions"

1b-iii) Open vs. closed, web-based (self-assessment) vs. face-to-face assessments in the METHODS section of the ABSTRACT

Mention how participants were recruited (online vs. offline), e.g., from an open access website or from a clinic or a closed online user group (closed usergroup trial), and clarify if this was a purely web-based trial, or there were face-to-face components (as part of the intervention or for assessment). Clearly say if outcomes were self-assessed through questionnaires (as common in web-based trials). Note: In traditional offline trials, an open trial (open-label trial) is a type of clinical trial in which both the researchers and participants know which treatment is being administered. To avoid confusion, use “blinded” or “unblinded” to indicated the level of blinding instead of “open”, as “open” in web-based trials usually refers to “open access” (i.e. participants can self-enrol). (Note: Only report in the abstract what the main paper is reporting. If this information is missing from the main body of text, consider adding it)

	1	2	3	4	5	
subitem not at all important	○	○	○	○	◉	essential

Does your paper address subitem 1b-iii?

Copy and paste relevant sections from the manuscript abstract (include quotes in quotation marks "like this" to indicate direct quotes from your manuscript), or elaborate on this item by providing additional information not in the ms, or briefly explain why the item is not applicable/relevant for your study

"...to receive 11 fully automated CBM training sessions"

1b-iv) RESULTS section in abstract must contain use data

Report number of participants enrolled/assessed in each group, the use/uptake of the intervention (e.g., attrition/adherence metrics, use over time, number of logins etc.), in addition to primary/secondary outcomes. (Note: Only report in the abstract what the main paper is reporting. If this information is missing from the main body of text, consider adding it)

	1	2	3	4	5	
subitem not at all important	○	○	○	◉	○	essential

Does your paper address subitem 1b-iv?

Copy and paste relevant sections from the manuscript abstract (include quotes in quotation marks "like this" to indicate direct quotes from your manuscript), or elaborate on this item by providing additional information not in the ms, or briefly explain why the item is not applicable/relevant for your study

"Only 10.7% (54/504) of the participants completed all 11 training sessions, and 8.3% (42/504) of the participants reached the 3-month follow-up assessment."

1b-v) CONCLUSIONS/DISCUSSION in abstract for negative trials

Conclusions/Discussions in abstract for negative trials: Discuss the primary outcome - if the trial is negative (primary outcome not changed), and the intervention was not used, discuss whether negative results are attributable to lack of uptake and discuss reasons. (Note: Only report in the abstract what the main paper is reporting. If this information is missing from the main body of text, consider adding it)

	1	2	3	4	5	
subitem not at all important	○	○	○	◉	○	essential

Does your paper address subitem 1b-v?

Copy and paste relevant sections from the manuscript abstract (include quotes in quotation marks "like this" to indicate direct quotes from your manuscript), or elaborate on this item by providing additional information not in the ms, or briefly explain why the item is not applicable/relevant for your study

"This RCT provides no support for the effectiveness of combining AtBM and ApBM in a self-help Web-based smoking cessation intervention. However, this study has a very high dropout rate and a very low frequency of training usage, indicating a general low acceptability of the intervention, which precludes any definite conclusion on its efficacy. We discuss how this study can inform future designs and settings of online CBM interventions."

INTRODUCTION

2a) In INTRODUCTION: Scientific background and explanation of rationale

2a-i) Problem and the type of system/solution

Describe the problem and the type of system/solution that is object of the study: intended as stand-alone intervention vs. incorporated in broader health care program? Intended for a particular patient population? Goals of the intervention, e.g., being more cost-effective to other interventions, replace or complement other solutions? (Note: Details about the intervention are provided in “Methods” under 5)

	1	2	3	4	5	
subitem not at all important	○	○	○	○	●	essential

Does your paper address subitem 2a-i? *

Copy and paste relevant sections from the manuscript (include quotes in quotation marks "like this" to indicate direct quotes from your manuscript), or elaborate on this item by providing additional information not in the ms, or briefly explain why the item is not applicable/relevant for your study

Problems:
"In the pioneering studies referenced above, the interventions only targeted one cognitive bias. Addictive behaviors, however, are characterized by multiple relatively independent cognitive biases [23]."

"Note that, at the time we set up this study (ie, in 2013), there was only one protocol study combining different Web-based CBM varieties in an intervention targeting alcohol use disorder [24], and no studies had yet investigated the effectiveness of a combined Web-based CBM intervention for smokers."

"In sum, evidence for the effectiveness of AtBM and ApBM in the smoking addiction domain is mixed."

Solutions:
"Thus, combining multiple CBM varieties that target different cognitive biases may enhance the treatment outcomes by combining their effects, as well as through potential synergistic effects."

"A key advantage of CBM interventions is that they are delivered as computerized tasks, which are easily administered online, featuring CBM as a potential easy-access and low-cost online intervention, particularly for the smoker population. Instead of attending formal smoking cessation programs [20], smokers often search for online help to quit smoking [21,22]. Therefore, we designed a Web-based CBM intervention specifically for smokers seeking self-help or help online."

"This study aimed at investigating the individual and combined effects of two varieties of Web-based CBM, AtBM and ApBM, in adult smokers who were seeking online help for quitting smoking."

2a-ii) Scientific background, rationale: What is known about the (type of) system

Scientific background, rationale: What is known about the (type of) system that is the object of the study (be sure to discuss the use of similar systems for other conditions/diagnoses, if appropiate), motivation for the study, i.e. what are the reasons for and what is the context for this specific study, from which stakeholder viewpoint is the study performed, potential impact of findings [2]. Briefly justify the choice of the comparator.

	1	2	3	4	5	
subitem not at all important	○	○	○	○	◉	essential

Does your paper address subitem 2a-ii? *

Copy and paste relevant sections from the manuscript (include quotes in quotation marks "like this" to indicate direct quotes from your manuscript), or elaborate on this item by providing additional information not in the ms, or briefly explain why the item is not applicable/relevant for your study

"Since 2013, some studies have explored the clinical effects of CBM as a behavior change intervention for smoking problems, although the evidence is still limited (see Mühlig et al [25] for a narrative review and Boffo et al [26] for a Bayesian meta-analysis). Three RCTs delivered CBM in an online setting, and five in a clinical or laboratory setting. Regarding the CBM studies in online settings, one RCT showed that Web-based ApBM alone could produce specific effects on reducing smoking in adult smokers [27], while two other RCTs did not support that multiple sessions of Web-based ApBM alone [28] or Web-based AtBM alone [11] were effective in promoting smoking cessation in adult smokers, although in the latter study, AtBM positively affected continued abstinence at the 6-month follow-up in a subgroup of heavy smokers [11]. Regarding the CBM studies in clinical or laboratory settings, one RCT showed that multiple sessions of ApBM plus CBT led to larger reductions in daily cigarette consumption in inpatient psychiatric smokers than sham training [29], while four other RCTs reported that multiple sessions of ApBM plus CBT [30,31], AtBM alone [32], or plus nicotine patches and behavioral support [33] did not result in better smoking treatment outcomes than sham training in smokers who intended to quit. In sum, evidence for the effectiveness of AtBM and ApBM in the smoking addiction domain is mixed. Therefore, more research is needed to investigate whether AtBM together with ApBM can benefit smoking cessation interventions."

2b) In INTRODUCTION: Specific objectives or hypotheses

Does your paper address CONSORT subitem 2b? *

Copy and paste relevant sections from the manuscript (include quotes in quotation marks "like this" to indicate direct quotes from your manuscript), or elaborate on this item by providing additional information not in the ms, or briefly explain why the item is not applicable/relevant for your study

Objectives:
"This study aimed at investigating the individual and combined effects of two varieties of Web-based CBM, AtBM and ApBM, in adult smokers who were seeking online help for quitting smoking."

Hypotheses:
"We hypothesized that, compared with its respective sham training, each type of active CBM training would (1) be more effective in fostering PPA and in decreasing DCU, and (2) lead to larger decreases in the specific cognitive bias it targeted. Given that AtBM and ApBM may tap into separate process [23], we also hypothesized that (3) the condition with double active CBM trainings would be the most effective in changing smoking-related outcomes."

METHODS

3a) Description of trial design (such as parallel, factorial) including allocation ratio

Does your paper address CONSORT subitem 3a? *

Copy and paste relevant sections from the manuscript (include quotes in quotation marks "like this" to indicate direct quotes from your manuscript), or elaborate on this item by providing additional information not in the ms, or briefly explain why the item is not applicable/relevant for your study

"A double blind RCT was conducted with a 2 × 2 factorial design, in which participants received 11 fully automated sessions of either an active or a sham version of both types of CBM training, resulting in four experimental conditions (active-AtBM + active-ApBM; active-AtBM+ sham-ApBM; sham-AtBM + active-ApBM; sham-AtBM + sham-ApBM; see Figure 1)."

"...participants were automatically randomized to one of the four training conditions by a computer randomization algorithm. The randomization was stratified by gender with a 1:1:1:1 ratio; therefore, participants were randomly assigned to one of the conditions to which the fewest participants of their gender had been allocated so far."

3b) Important changes to methods after trial commencement (such as eligibility criteria), with reasons

Does your paper address CONSORT subitem 3b? *

Copy and paste relevant sections from the manuscript (include quotes in quotation marks "like this" to indicate direct quotes from your manuscript), or elaborate on this item by providing additional information not in the ms, or briefly explain why the item is not applicable/relevant for your study

"Note that the primary outcome preregistered was 7-day PPA at the follow-up assessments. That is, in the original plan, we focused on the medium-term training effects on the smoking status. However, because of the huge dropout rates (see Figure 1), we decided to include all available data at all assessments. As a result, in the current report, we focused on the changes in the PPA over time."

3b-i) Bug fixes, Downtimes, Content Changes

Bug fixes, Downtimes, Content Changes: ehealth systems are often dynamic systems. A description of changes to methods therefore also includes important changes made on the intervention or comparator during the trial (e.g., major bug fixes or changes in the functionality or content) (5-iii) and other "unexpected events" that may have influenced study design such as staff changes, system failures/downtimes, etc. [2].

	1	2	3	4	5	
subitem not at all important	○	○	◉	○	○	essential

Does your paper address subitem 3b-i?

Copy and paste relevant sections from the manuscript (include quotes in quotation marks "like this" to indicate direct quotes from your manuscript), or elaborate on this item by providing additional information not in the ms, or briefly explain why the item is not applicable/relevant for your study

There were no big bugs or any changes of the intervention content after the trial launched. Small technical problems during the training were fixed by an IT staff in our department.

"Acknowledgments:
The authors would like to thank Bruno Boutin for his contributions to the technical setup

4a) Eligibility criteria for participants

Does your paper address CONSORT subitem 4a? *

Copy and paste relevant sections from the manuscript (include quotes in quotation marks "like this" to indicate direct quotes from your manuscript), or elaborate on this item by providing additional information not in the ms, or briefly explain why the item is not applicable/relevant for your study

"As the study concerned a self-help intervention open to everybody, there were no specific inclusion criteria, except for being 18 years and older and able to understand Dutch (the intervention was only provided in Dutch)."

4a-i) Computer / Internet literacy

Computer / Internet literacy is often an implicit “de facto” eligibility criterion - this should be explicitly clarified.

	1	2	3	4	5	
subitem not at all important	○	◉	○	○	○	essential

Does your paper address subitem 4a-i?

Copy and paste relevant sections from the manuscript (include quotes in quotation marks "like this" to indicate direct quotes from your manuscript), or elaborate on this item by providing additional information not in the ms, or briefly explain why the item is not applicable/relevant for your study

Your answer

4a-ii) Open vs. closed, web-based vs. face-to-face assessments:

Open vs. closed, web-based vs. face-to-face assessments: Mention how participants were recruited (online vs. offline), e.g., from an open access website or from a clinic, and clarify if this was a purely web-based trial, or there were face-to-face components (as part of the intervention or for assessment), i.e., to what degree got the study team to know the participant. In online-only trials, clarify if participants were quasi-anonymous and whether having multiple identities was possible or whether technical or logistical measures (e.g., cookies, email confirmation, phone calls) were used to detect/prevent these.

	1	2	3	4	5	
subitem not at all important	○	○	○	◉	○	essential

Does your paper address subitem 4a-ii? *

Copy and paste relevant sections from the manuscript (include quotes in quotation marks "like this" to indicate direct quotes from your manuscript), or elaborate on this item by providing additional information not in the ms, or briefly explain why the item is not applicable/relevant for your study

"Adult smokers were recruited across the Netherlands through our lab website (Addiction Development and Psychopathology Lab of the University of Amsterdam; ADAPT [37]), press releases (eg, TV interviews, newspapers, and scientific books [38]), and word-of-mouth communication. The ADAPT website is open-access and provided a series of cognitive training targeting variety of addition and affective-related problems such as smoking, alcohol, gambling, anxiety, and depression."

"All training session were Web-based and all assessment sessions were self-assessed via Web-based questionnaires and computerized tasks."

"Participants could contact the responsible researcher (the second author) by email in case they had questions or technical problems."

"Since the enrollment occurred online, additional actions were taken to check multiple identities. Participants who used similar usernames, email addresses, and internet protocol addresses were excluded in the data analysis stage (n=8)."

4a-iii) Information giving during recruitment

Information given during recruitment. Specify how participants were briefed for recruitment and in the informed consent procedures (e.g., publish the informed consent documentation as appendix, see also item X26), as this information may have an effect on user self-selection, user expectation and may also bias results.

	1	2	3	4	5	
subitem not at all important	○	○	○	◉	○	essential

Does your paper address subitem 4a-iii?

Copy and paste relevant sections from the manuscript (include quotes in quotation marks "like this" to indicate direct quotes from your manuscript), or elaborate on this item by providing additional information not in the ms, or briefly explain why the item is not applicable/relevant for your study

"Interested participants were directed to the study website [39]. The website explained the scientific rationale of CBM training, training to overcome mental habits and automatic responses to smoking, and explained that the current intervention program combined two types of CBM freely available for people who would like to quit smoking."

"Through the consent form, participants were fully informed about the whole study procedure, that the effectiveness of the two CBM trainings was being tested (compared with two training types where no or smaller effects were expected, ie, sham training), and that they had a 25% chance to be assigned to the condition with two sham trainings. "

4b) Settings and locations where the data were collected

Does your paper address CONSORT subitem 4b? *

Copy and paste relevant sections from the manuscript (include quotes in quotation marks "like this" to indicate direct quotes from your manuscript), or elaborate on this item by providing additional information not in the ms, or briefly explain why the item is not applicable/relevant for your study

"Adult smokers were recruited across the Netherlands through our lab website (Addiction Development and Psychopathology Lab of the University of Amsterdam; ADAPT [37]), press releases (eg, TV interviews, newspapers, and scientific books [38]), and word-of-mouth communication."

4b-i) Report if outcomes were (self-)assessed through online questionnaires

Clearly report if outcomes were (self-)assessed through online questionnaires (as common in web-based trials) or otherwise.

	1	2	3	4	5	
subitem not at all important	○	○	○	●	○	essential

Does your paper address subitem 4b-i? *

Copy and paste relevant sections from the manuscript (include quotes in quotation marks "like this" to indicate direct quotes from your manuscript), or elaborate on this item by providing additional information not in the ms, or briefly explain why the item is not applicable/relevant for your study

"...all assessment sessions were self-assessed via Web-based questionnaires and computerized tasks."

4b-ii) Report how institutional affiliations are displayed

Report how institutional affiliations are displayed to potential participants [on ehealth media], as affiliations with prestigious hospitals or universities may affect volunteer rates, use, and reactions with regards to an intervention.(Not a required item – describe only if this may bias results)

	1	2	3	4	5	
subitem not at all important	○	○	●	○	○	essential

Does your paper address subitem 4b-ii?

Copy and paste relevant sections from the manuscript (include quotes in quotation marks "like this" to indicate direct quotes from your manuscript), or elaborate on this item by providing additional information not in the ms, or briefly explain why the item is not applicable/relevant for your study

"The ADAPT website is open-access and provided a series of cognitive training targeting variety of addition and affective-related problems such as smoking, alcohol, gambling, anxiety, and depression."

5) The interventions for each group with sufficient details to allow replication, including how and when they were actually administered

5-i) Mention names, credential, affiliations of the developers, sponsors, and owners

Mention names, credential, affiliations of the developers, sponsors, and owners [6] (if authors/evaluators are owners or developer of the software, this needs to be declared in a “Conflict of interest” section or mentioned elsewhere in the manuscript).

	1	2	3	4	5	
subitem not at all important	○	○	○	●	○	essential

Does your paper address subitem 5-i?

Copy and paste relevant sections from the manuscript (include quotes in quotation marks "like this" to indicate direct quotes from your manuscript), or elaborate on this item by providing additional information not in the ms, or briefly explain why the item is not applicable/relevant for your study

"To re-train AtB, we used a modified version of the VPT [24,40]."

"To retrain ApB, we used a modified version of the AAT [24,40]."

Acknowledgements:
"The authors would like to thank Bruno Boutin for his contributions to the technical setup and maintenance of the website of this study."

5-ii) Describe the history/development process

Describe the history/development process of the application and previous formative evaluations (e.g., focus groups, usability testing), as these will have an impact on adoption/use rates and help with interpreting results.

	1	2	3	4	5	
subitem not at all important	○	○	●	○	○	essential

Does your paper address subitem 5-ii?

Copy and paste relevant sections from the manuscript (include quotes in quotation marks "like this" to indicate direct quotes from your manuscript), or elaborate on this item by providing additional information not in the ms, or briefly explain why the item is not applicable/relevant for your study

"To re-train AtB, we used a modified version of the VPT [24,40]."

"To retrain ApB, we used a modified version of the AAT [24,40]."

5-iii) Revisions and updating

Revisions and updating. Clearly mention the date and/or version number of the application/intervention (and comparator, if applicable) evaluated, or describe whether the intervention underwent major changes during the evaluation process, or whether the development and/or content was "frozen" during the trial. Describe dynamic components such as news feeds or changing content which may have an impact on the replicability of the intervention (for unexpected events see item 3b).

	1	2	3	4	5	
subitem not at all important	○	○	●	○	○	essential

Does your paper address subitem 5-iii?

Copy and paste relevant sections from the manuscript (include quotes in quotation marks "like this" to indicate direct quotes from your manuscript), or elaborate on this item by providing additional information not in the ms, or briefly explain why the item is not applicable/relevant for your study

N/A.

5-iv) Quality assurance methods

Provide information on quality assurance methods to ensure accuracy and quality of information provided [1], if applicable.

	1	2	3	4	5	
subitem not at all important	○	○	●	○	○	essential

Does your paper address subitem 5-iv?

Copy and paste relevant sections from the manuscript (include quotes in quotation marks "like this" to indicate direct quotes from your manuscript), or elaborate on this item by providing additional information not in the ms, or briefly explain why the item is not applicable/relevant for your study

"Bootstrapped split-half reliability estimates [44] for both VPT and AAT at baseline were obtained by using the splithalf package in R (version 0.3.1 [45]), which performed 5000 random splits. The reliability of VPT was r=0.25, 95% CI 0.19 to 0.31 (Spearman-Brown corrected rsb=0.40, 95% CI 0.32 to 0.48), and the reliability of AAT was r=0.02, 95% CI −0.10 to 0.13 (Spearman-Brown corrected rsb=0.03, 95% CI −0.18 to 0.23)."

5-v) Ensure replicability by publishing the source code, and/or providing screenshots/screen-capture video, and/or providing flowcharts of the algorithms used

Ensure replicability by publishing the source code, and/or providing screenshots/screen-capture video, and/or providing flowcharts of the algorithms used. Replicability (i.e., other researchers should in principle be able to replicate the study) is a hallmark of scientific reporting.

	1	2	3	4	5	
subitem not at all important	○	○	○	◉	○	essential

Does your paper address subitem 5-v?

Copy and paste relevant sections from the manuscript (include quotes in quotation marks "like this" to indicate direct quotes from your manuscript), or elaborate on this item by providing additional information not in the ms, or briefly explain why the item is not applicable/relevant for your study

Study websites were provided:
"Adult smokers were recruited across the Netherlands through our lab website (Addiction Development and Psychopathology Lab of the University of Amsterdam; ADAPT [37])..."

"Interested participants were directed to the study website [39]."

5-vi) Digital preservation

Digital preservation: Provide the URL of the application, but as the intervention is likely to change or disappear over the course of the years; also make sure the intervention is archived (Internet Archive, webcitation.org, and/or publishing the source code or screenshots/videos alongside the article). As pages behind login screens cannot be archived, consider creating demo pages which are accessible without login.

	1	2	3	4	5	
subitem not at all important	○	○	○	●	○	essential

Does your paper address subitem 5-vi?

Copy and paste relevant sections from the manuscript (include quotes in quotation marks "like this" to indicate direct quotes from your manuscript), or elaborate on this item by providing additional information not in the ms, or briefly explain why the item is not applicable/relevant for your study

References:
"37. Addiction Development and Psycholopathology (ADAPT) lab website, University of Amsterdam. Available from: http://www.impliciet.eu/. Archived at: http://www.webcitation.org/77YbQbruP"

"The study website, University of Amsterdam. Available from: http://www.impliciet.eu/trainings/roken/. Archived at: http://www.webcitation.org/77YbyMQgz"

5-vii) Access

Access: Describe how participants accessed the application, in what setting/context, if they had to pay (or were paid) or not, whether they had to be a member of specific group. If known, describe how participants obtained "access to the platform and Internet" [1]. To ensure access for editors/reviewers/readers, consider to provide a "backdoor" login account or demo mode for reviewers/readers to explore the application (also important for archiving purposes, see vi).

	1	2	3	4	5	
subitem not at all important	○	○	○	●	○	essential

Does your paper address subitem 5-vii? *

Copy and paste relevant sections from the manuscript (include quotes in quotation marks "like this" to indicate direct quotes from your manuscript), or elaborate on this item by providing additional information not in the ms, or briefly explain why the item is not applicable/relevant for your study

"Adult smokers were recruited across the Netherlands through our lab website (Addiction Development and Psychopathology Lab of the University of Amsterdam; ADAPT [37]), press releases (eg, TV interviews, newspapers, and scientific books [38]), and word-of-mouth communication. The ADAPT website is open-access and provided a series of cognitive training targeting variety of addition and affective-related problems such as smoking, alcohol, gambling, anxiety, and depression."

"Interested participants were directed to the study website [39]."

"Upon registration, participants created their own user account by providing their username, password, and email address, were screened regarding their age and were then randomized (see the Randomization and Blinding section). Participants were notified of their eligibility via email. Eligible participants who clicked on their emailed link were returned to the study website where they submitted a consent form."

5-viii) Mode of delivery, features/functionalities/components of the intervention and comparator, and the theoretical framework

Describe mode of delivery, features/functionalities/components of the intervention and comparator, and the theoretical framework [6] used to design them (instructional strategy [1], behaviour change techniques, persuasive features, etc., see e.g., [7, 8] for terminology). This includes an in-depth description of the content (including where it is coming from and who developed it) [1],” whether [and how] it is tailored to individual circumstances and allows users to track their progress and receive feedback” [6]. This also includes a description of communication delivery channels and – if computer-mediated communication is a component – whether communication was synchronous or asynchronous [6]. It also includes information on presentation strategies [1], including page design principles, average amount of text on pages, presence of hyperlinks to other resources, etc. [1].

	1	2	3	4	5	
subitem not at all important	○	○	○	◉	○	essential

Does your paper address subitem 5-viii? *

Copy and paste relevant sections from the manuscript (include quotes in quotation marks "like this" to indicate direct quotes from your manuscript), or elaborate on this item by providing additional information not in the ms, or briefly explain why the item is not applicable/relevant for your study

"Attentional Bias Modification
To re-train AtB, we used a modified version of the VPT [24,40]. Participants in the active training condition were trained to shift their attention from smoking-related pictures to neutral pictures by exposing them only to nonsmoking trials (ie, the probe only followed the neutral pictures), whereas participants in the sham training condition were presented with 50% smoking trials and 50% nonsmoking trials (ie, continued assessment). Each AtBM session started with a practice block (8 trials) and a mini-assessment block (128 trials), after which, participants received the active or sham version of the AtBM (192 trials)."

"Approach Bias Modification
To retrain ApB, we used a modified version of the AAT [24,40]. Participants in the active training condition were trained to avoid smoking-related pictures by exposing them only to smoking/push and neutral/pull trials, whereas participants in the sham training condition were presented with 50% pull and 50% push trials for both smoking-related and neutral pictures (ie, continues assessment). Each ApBM session started with a practice block (12 trials) and a mini-assessment block (64 trials), after which, participants received the active or sham version of the ApBM (192 trials)."

5-ix) Describe use parameters

Describe use parameters (e.g., intended “doses” and optimal timing for use). Clarify what instructions or recommendations were given to the user, e.g., regarding timing, frequency, heaviness of use, if any, or was the intervention used ad libitum.

	1	2	3	4	5	
subitem not at all important	○	○	○	◉	○	essential

Does your paper address subitem 5-ix?

Copy and paste relevant sections from the manuscript (include quotes in quotation marks "like this" to indicate direct quotes from your manuscript), or elaborate on this item by providing additional information not in the ms, or briefly explain why the item is not applicable/relevant for your study

"Participants were suggested to complete the 11 training sessions within 4 weeks (eg, three sessions per week). Participants were allowed to train on a daily basis and could arrange their own training schedule."

5-x) Clarify the level of human involvement

Clarify the level of human involvement (care providers or health professionals, also technical assistance) in the e-intervention or as co-intervention (detail number and expertise of professionals involved, if any, as well as “type of assistance offered, the timing and frequency of the support, how it is initiated, and the medium by which the assistance is delivered”. It may be necessary to distinguish between the level of human involvement required for the trial, and the level of human involvement required for a routine application outside of a RCT setting (discuss under item 21 – generalizability).

	1	2	3	4	5	
subitem not at all important	○	○	○	◉	○	essential

Does your paper address subitem 5-x?

Copy and paste relevant sections from the manuscript (include quotes in quotation marks "like this" to indicate direct quotes from your manuscript), or elaborate on this item by providing additional information not in the ms, or briefly explain why the item is not applicable/relevant for your study

"All training session were Web-based and all assessment sessions were self-assessed via Web-based questionnaires and computerized tasks."

"Participants could contact the responsible researcher (the second author) by email in case they had questions or technical problems."

"All participants received a brief automated tailored feedback at the beginning of the baseline assessment."

5-xi) Report any prompts/reminders used

Report any prompts/reminders used: Clarify if there were prompts (letters, emails, phone calls, SMS) to use the application, what triggered them, frequency etc. It may be necessary to distinguish between the level of prompts/reminders required for the trial, and the level of prompts/reminders for a routine application outside of a RCT setting (discuss under item 21 – generalizability).

	1	2	3	4	5	
subitem not at all important	○	○	○	◉	○	essential

Does your paper address subitem 5-xi? *

Copy and paste relevant sections from the manuscript (include quotes in quotation marks "like this" to indicate direct quotes from your manuscript), or elaborate on this item by providing additional information not in the ms, or briefly explain why the item is not applicable/relevant for your study

"Each training or assessment session automatically opened 24 hours after the previous session was completed and stayed open for 30 days. When each session opened, an automated notification was sent to the participants. If participants did not complete the session, they received an automated reminder email after 3 days, 7 days, 11 days, and 3 weeks until the session closed (including the baseline session)."

5-xii) Describe any co-interventions (incl. training/support)

Describe any co-interventions (incl. training/support): Clearly state any interventions that are provided in addition to the targeted eHealth intervention, as ehealth intervention may not be designed as stand-alone intervention. This includes training sessions and support [1]. It may be necessary to distinguish between the level of training required for the trial, and the level of training for a routine application outside of a RCT setting (discuss under item 21 – generalizability.

	1	2	3	4	5	
subitem not at all important	○	○	○	◉	○	essential

Does your paper address subitem 5-xii? *

Copy and paste relevant sections from the manuscript (include quotes in quotation marks "like this" to indicate direct quotes from your manuscript), or elaborate on this item by providing additional information not in the ms, or briefly explain why the item is not applicable/relevant for your study

"To increase participants' intrinsic motivation to change their smoking behaviors before the CBM training, all participants first received a brief, automated, tailored feedback, irrespective of their CBM condition."

"Automated Tailored Feedback
All participants received a brief automated tailored feedback at the beginning of the baseline assessment. This session provided feedback based on participants' current smoking behaviors, attitudes toward smoking, perceived importance, confidence, and motivation to quit, and goals and plans to change smoking behaviors [41,42]. The tailored feedback consisted of (1) summarizing the information participants provided, (2) comparing their smoking behaviors and attitudes toward smoking with current smokers and ex-smokers, (3) challenging and modifying their positive attitudes toward smoking by providing health risk information, and (4) providing tips and supports for their further changing progress."

6a) Completely defined pre-specified primary and secondary outcome measures, including how and when they were assessed

Does your paper address CONSORT subitem 6a? *

Copy and paste relevant sections from the manuscript (include quotes in quotation marks "like this" to indicate direct quotes from your manuscript), or elaborate on this item by providing additional information not in the ms, or briefly explain why the item is not applicable/relevant for your study

"The primary outcome PPA was determined by using the timeline follow back method (TLFB [43]) and was assessed at six main assessment time points (eg, baseline, midtraining, posttraining, and follow-ups at 1, 2, and 3 months) and 11 interim assessment time points (eg, a brief assessment before each training session)."

"The secondary behavioral outcome DCU was also derived from the TLFB data."

"Secondary cognitive outcomes included AtB and ApB assessed with the online version of the VPT and AAT, respectively, described above (see the Cognitive Bias Modification section). Both biases were assessed at four main assessment time points (eg, baseline, midtraining, posttraining, and 3-month follow-up) and 11 interim assessment time points (eg, a mini-assessment block in each training session)."

"Since craving, depression severity, and motivation to quit smoking have been found to be related to cognitive biases or smoking behaviors [34-36], these variables were also included in this study as additional secondary outcomes...The methods and results for the additional secondary outcomes and the exploratory moderation analyses are reported in the Multimedia Appendices 1 and 2."

6a-i) Online questionnaires: describe if they were validated for online use and apply CHERRIES items to describe how the questionnaires were designed/deployed

If outcomes were obtained through online questionnaires, describe if they were validated for online use and apply CHERRIES items to describe how the questionnaires were designed/deployed [9].

	1	2	3	4	5	
subitem not at all important	○	○	◉	○	○	essential

Does your paper address subitem 6a-i?

Copy and paste relevant sections from manuscript text

Your answer

6a-ii) Describe whether and how “use” (including intensity of use/dosage) was defined/measured/monitored

Describe whether and how “use” (including intensity of use/dosage) was defined/measured/monitored (logins, logfile analysis, etc.). Use/adoption metrics are important process outcomes that should be reported in any ehealth trial.

	1	2	3	4	5	
subitem not at all important	○	○	◉	○	○	essential

Does your paper address subitem 6a-ii?

Copy and paste relevant sections from manuscript text

Your answer

6a-iii) Describe whether, how, and when qualitative feedback from participants was obtained

Describe whether, how, and when qualitative feedback from participants was obtained (e.g., through emails, feedback forms, interviews, focus groups).

	1	2	3	4	5	
subitem not at all important	○	○	○	◉	○	essential

Does your paper address subitem 6a-iii?

Copy and paste relevant sections from manuscript text

"The training evaluation questions (TEQ) were administrated at the posttraining assessment, where participants evaluated both the CBM training as a whole and the AtBM and ApBM training, respectively...For participants dropping out of the study before completing the posttraining assessment, the TEQ could be triggered by the participants themselves when requiring to stop the study, by clicking on a Web link included in the reminder emails they received."

6b) Any changes to trial outcomes after the trial commenced, with reasons

Does your paper address CONSORT subitem 6b? *

Copy and paste relevant sections from the manuscript (include quotes in quotation marks "like this" to indicate direct quotes from your manuscript), or elaborate on this item by providing additional information not in the ms, or briefly explain why the item is not applicable/relevant for your study

"Note that the primary outcome preregistered was 7-day PPA at the follow-up assessments. That is, in the original plan, we focused on the medium-term training effects on the smoking status. However, because of the huge dropout rates (see Figure 1), we decided to include all available data at all assessments. As a result, in the current report, we focused on the changes in the PPA over time."

7a) How sample size was determined

NPT: When applicable, details of whether and how the clustering by care provides or centers was addressed

7a-i) Describe whether and how expected attrition was taken into account when calculating the sample size

Describe whether and how expected attrition was taken into account when calculating the sample size.

	1	2	3	4	5	
subitem not at all important	○	○	○	●	○	essential

Does your paper address subitem 7a-i?

Copy and paste relevant sections from manuscript title (include quotes in quotation marks "like this" to indicate direct quotes from your manuscript), or elaborate on this item by providing additional information not in the ms, or briefly explain why the item is not applicable/relevant for your study

"Since this was the first study to test the effectiveness of two combined Web-based CBM trainings as a behavior change intervention for smoking cessation, no effective knowledge (eg, CBM training effect sizes and dropout rates) was available for us to calculate the sample size at the time we set up the study. Therefore, our aim was to recruit as many participants as possible with the minimum of at least 75 participants per training condition."

7b) When applicable, explanation of any interim analyses and stopping guidelines

Does your paper address CONSORT subitem 7b? *

Copy and paste relevant sections from the manuscript (include quotes in quotation marks "like this" to indicate direct quotes from your manuscript), or elaborate on this item by providing additional information not in the ms, or briefly explain why the item is not applicable/relevant for your study

"We stopped the recruitment in December 2018."

8a) Method used to generate the random allocation sequence

NPT: When applicable, how care providers were allocated to each trial group

Does your paper address CONSORT subitem 8a? *

Copy and paste relevant sections from the manuscript (include quotes in quotation marks "like this" to indicate direct quotes from your manuscript), or elaborate on this item by providing additional information not in the ms, or briefly explain why the item is not applicable/relevant for your study

"Upon creation of a study account, participants were automatically randomized to one of the four training conditions by a computer randomization algorithm. The randomization was stratified by gender with a 1:1:1:1 ratio; therefore, participants were randomly assigned to one of the conditions to which the fewest participants of their gender had been allocated so far."

8b) Type of randomisation; details of any restriction (such as blocking and block size)

Does your paper address CONSORT subitem 8b? *

Copy and paste relevant sections from the manuscript (include quotes in quotation marks "like this" to indicate direct quotes from your manuscript), or elaborate on this item by providing additional information not in the ms, or briefly explain why the item is not applicable/relevant for your study

"The randomization was stratified by gender with a 1:1:1:1 ratio; therefore, participants were randomly assigned to one of the conditions to which the fewest participants of their gender had been allocated so far."

9) Mechanism used to implement the random allocation sequence (such as sequentially numbered containers), describing any steps taken to conceal the sequence until interventions were assigned

Does your paper address CONSORT subitem 9? *

Copy and paste relevant sections from the manuscript (include quotes in quotation marks "like this" to indicate direct quotes from your manuscript), or elaborate on this item by providing additional information not in the ms, or briefly explain why the item is not applicable/relevant for your study

"The randomization was stratified by gender with a 1:1:1:1 ratio; therefore, participants were randomly assigned to one of the conditions to which the fewest participants of their gender had been allocated so far."

10) Who generated the random allocation sequence, who enrolled participants, and who assigned participants to interventions

Does your paper address CONSORT subitem 10? *

Copy and paste relevant sections from the manuscript (include quotes in quotation marks "like this" to indicate direct quotes from your manuscript), or elaborate on this item by providing additional information not in the ms, or briefly explain why the item is not applicable/relevant for your study

"Upon creation of a study account, participants were automatically randomized to one of the four training conditions by a computer randomization algorithm."

11a) If done, who was blinded after assignment to interventions (for example, participants, care providers, those assessing outcomes) and how

NPT: Whether or not administering co-interventions were blinded to group assignment

11a-i) Specify who was blinded, and who wasn't

Specify who was blinded, and who wasn't. Usually, in web-based trials it is not possible to blind the participants [1, 3] (this should be clearly acknowledged), but it may be possible to blind outcome assessors, those doing data analysis or those administering co-interventions (if any).

	1	2	3	4	5	
subitem not at all important	○	○	○	○	◉	essential

Does your paper address subitem 11a-i? *

Copy and paste relevant sections from the manuscript (include quotes in quotation marks "like this" to indicate direct quotes from your manuscript), or elaborate on this item by providing additional information not in the ms, or briefly explain why the item is not applicable/relevant for your study

"The study was as a double-blind trial...Since the randomization was fully automated and conducted by a computer algorithm, allocation concealment was ensured. In addition, the automated study procedure ensured that participants were blinded to the training condition they were assigned to. The second author could access the database during the data collection to monitor the data collection process and was responsible to reply to participants in case they had questions and/oror technical problems. The first author could access the database after data collection completion to download and analyze the data. No authors provided any treatment to the participants, nor assessed any of the outcomes during the trial."

11a-ii) Discuss e.g., whether participants knew which intervention was the "intervention of interest" and which one was the "comparator"

Informed consent procedures (4a-ii) can create biases and certain expectations - discuss e.g., whether participants knew which intervention was the "intervention of interest" and which one was the "comparator".

	1	2	3	4	5	
subitem not at all important	○	○	○	○	◉	essential

Does your paper address subitem 11a-ii?

Copy and paste relevant sections from the manuscript (include quotes in quotation marks "like this" to indicate direct quotes from your manuscript), or elaborate on this item by providing additional information not in the ms, or briefly explain why the item is not applicable/relevant for your study

"Through the consent form, participants were fully informed about the whole study procedure, that the effectiveness of the two CBM trainings was being tested (compared with two training types where no or smaller effects were expected, ie, sham training), and that they had a 25% chance to be assigned to the condition with two sham trainings."

"In addition, the automated study procedure ensured that participants were blinded to the training condition they were assigned to."

11b) If relevant, description of the similarity of interventions

(this item is usually not relevant for ehealth trials as it refers to similarity of a placebo or sham intervention to a active medication/intervention)

Does your paper address CONSORT subitem 11b? *

Copy and paste relevant sections from the manuscript (include quotes in quotation marks "like this" to indicate direct quotes from your manuscript), or elaborate on this item by providing additional information not in the ms, or briefly explain why the item is not applicable/relevant for your study

"Attentional Bias Modification
To re-train AtB, we used a modified version of the VPT [24,40]. Participants in the active training condition were trained to shift their attention from smoking-related pictures to neutral pictures by exposing them only to nonsmoking trials (ie, the probe only followed the neutral pictures), whereas participants in the sham training condition were presented with 50% smoking trials and 50% nonsmoking trials (ie, continued assessment)."

"Approach Bias Modification
To retrain ApB, we used a modified version of the AAT [24,40]. Participants in the active training condition were trained to avoid smoking-related pictures by exposing them only to smoking/push and neutral/pull trials, whereas participants in the sham training condition were presented with 50% pull and 50% push trials for both smoking-related and neutral pictures (ie, continues assessment)."

12a) Statistical methods used to compare groups for primary and secondary outcomes

NPT: When applicable, details of whether and how the clustering by care providers or centers was addressed

Does your paper address CONSORT subitem 12a? *

Copy and paste relevant sections from the manuscript (include quotes in quotation marks "like this" to indicate direct quotes from your manuscript), or elaborate on this item by providing additional information not in the ms, or briefly explain why the item is not applicable/relevant for your study

"To test the training effects, a Multilevel Modeling (MLM) approach was adopted, which allows for intention-to-treat (ITT) analysis including all available data and takes the clustering of data by participants into account [49]. In our analyses, all models incorporated a random intercept for participants, used the maximum likelihood estimator, and were conducted in R with the lme4 (version 1.1.17 [50]) and lmerTest packages (version 3.0.1 [51]). An alpha of .05 (two-sided) was applied to all hypotheses testing.
We used a piecewise step-function growth curve model [52] to track outcome changes over time. For PPA, the training effects were evaluated over three time phases: first half intervention phase (TP1: from baseline to midtraining assessment), second half intervention phase (TP2: from interim assessment 6 to posttraining assessment), and follow-up phase (TP3: from interim assessment 11 to the 3-month follow-up). Although all participants were coded as 0 (not quit) for PPA at baseline, modeling the growth curve starting at baseline is necessary for ITT analyses. Therefore, baseline was included into TP1. For DCU, AtB, and ApB, the training effects were evaluated over four time phases: baseline (TP1), first half intervention phase (TP2: from interim assessment 1 to midtraining assessment), and, similar to PPA, second intervention phase (TP3), and follow-up phase (TP4).
To test the training effects on PPA, a multilevel logistic regression analysis was conducted, whereas for DCU, AtB, and ApB, a series of multilevel linear regression analyses was conducted. Predictors included time phase, AtBM (active vs sham), ApBM (active vs sham), and their interactions. Our hypotheses testing focused on the two-way interaction effects of time phase × AtBM and time phase × ApBM, and the three-way interaction effects of time phase × AtBM × ApBM."

12a-i) Imputation techniques to deal with attrition / missing values

Imputation techniques to deal with attrition / missing values: Not all participants will use the intervention/comparator as intended and attrition is typically high in ehealth trials. Specify how participants who did not use the application or dropped out from the trial were treated in the statistical analysis (a complete case analysis is strongly discouraged, and simple imputation techniques such as LOCF may also be problematic [4]).

	1	2	3	4	5	
subitem not at all important	○	○	○	◉	○	essential

Does your paper address subitem 12a-i? *

Copy and paste relevant sections from the manuscript (include quotes in quotation marks "like this" to indicate direct quotes from your manuscript), or elaborate on this item by providing additional information not in the ms, or briefly explain why the item is not applicable/relevant for your study

"To test the training effects, a Multilevel Modeling (MLM) approach was adopted, which allows for intention-to-treat (ITT) analysis including all available data and takes the clustering of data by participants into account [49]."

12b) Methods for additional analyses, such as subgroup analyses and adjusted analyses

Does your paper address CONSORT subitem 12b? *

Copy and paste relevant sections from the manuscript (include quotes in quotation marks "like this" to indicate direct quotes from your manuscript), or elaborate on this item by providing additional information not in the ms, or briefly explain why the item is not applicable/relevant for your study

"The methods and results for the additional secondary outcomes and the exploratory moderation analyses are reported in the Multimedia Appendices 1 and 2."

X26) REB/IRB Approval and Ethical Considerations [recommended as subheading under "Methods"] (not a CONSORT item)

X26-i) Comment on ethics committee approval

	1	2	3	4	5	
subitem not at all important	○	○	○	○	●	essential

Does your paper address subitem X26-i?

Copy and paste relevant sections from the manuscript (include quotes in quotation marks "like this" to indicate direct quotes from your manuscript), or elaborate on this item by providing additional information not in the ms, or briefly explain why the item is not applicable/relevant for your study

"The study was approved by the Ethics Committee of Psychology at the University of Amsterdam (reference number: 2013-DP-3047)..."

x26-ii) Outline informed consent procedures

Outline informed consent procedures e.g., if consent was obtained offline or online (how? Checkbox, etc.?), and what information was provided (see 4a-ii). See [6] for some items to be included in informed consent documents.

	1	2	3	4	5	
subitem not at all important	○	○	○	○	◉	essential

Does your paper address subitem X26-ii?

Copy and paste relevant sections from the manuscript (include quotes in quotation marks "like this" to indicate direct quotes from your manuscript), or elaborate on this item by providing additional information not in the ms, or briefly explain why the item is not applicable/relevant for your study

"Participants were notified of their eligibility via email. Eligible participants who clicked on their emailed link were returned to the study website where they submitted a consent form. Through the consent form, participants were fully informed about the whole study procedure, that the effectiveness of the two CBM trainings was being tested (compared with two training types where no or smaller effects were expected, ie, sham training), and that they had a 25% chance to be assigned to the condition with two sham trainings."

X26-iii) Safety and security procedures

Safety and security procedures, incl. privacy considerations, and any steps taken to reduce the likelihood or detection of harm (e.g., education and training, availability of a hotline)

	1	2	3	4	5	
subitem not at all important	○	○	◉	○	○	essential

Does your paper address subitem X26-iii?

Copy and paste relevant sections from the manuscript (include quotes in quotation marks "like this" to indicate direct quotes from your manuscript), or elaborate on this item by providing additional information not in the ms, or briefly explain why the item is not applicable/relevant for your study

"Participants could contact the responsible researcher (the second author) by email in case they had questions or technical problems."

RESULTS

13a) For each group, the numbers of participants who were randomly assigned, received intended treatment, and were analysed for the primary outcome

NPT: The number of care providers or centers performing the intervention in each group and the number of patients treated by each care provider in each center

Does your paper address CONSORT subitem 13a? *

Copy and paste relevant sections from the manuscript (include quotes in quotation marks "like this" to indicate direct quotes from your manuscript), or elaborate on this item by providing additional information not in the ms, or briefly explain why the item is not applicable/relevant for your study

"The Consolidated Standards of Reporting Trials (CONSORT) flow diagram is reported in Figure 1."

13b) For each group, losses and exclusions after randomisation, together with reasons

Does your paper address CONSORT subitem 13b? (NOTE: Preferably, this is shown in a CONSORT flow diagram) *

Copy and paste relevant sections from the manuscript (include quotes in quotation marks "like this" to indicate direct quotes from your manuscript), or elaborate on this item by providing additional information not in the ms, or briefly explain why the item is not applicable/relevant for your study

"The Consolidated Standards of Reporting Trials (CONSORT) flow diagram is reported in Figure 1."

13b-i) Attrition diagram

Strongly recommended: An attrition diagram (e.g., proportion of participants still logging in or using the intervention/comparator in each group plotted over time, similar to a survival curve) or other figures or tables demonstrating usage/dose/engagement.

	1	2	3	4	5	
subitem not at all important	○	○	○	◉	○	essential

Does your paper address subitem 13b-i?

Copy and paste relevant sections from the manuscript or cite the figure number if applicable (include quotes in quotation marks "like this" to indicate direct quotes from your manuscript), or elaborate on this item by providing additional information not in the ms, or briefly explain why the item is not applicable/relevant for your study

"The Consolidated Standards of Reporting Trials (CONSORT) flow diagram is reported in Figure 1."

14a) Dates defining the periods of recruitment and follow-up

Does your paper address CONSORT subitem 14a? *

Copy and paste relevant sections from the manuscript (include quotes in quotation marks "like this" to indicate direct quotes from your manuscript), or elaborate on this item by providing additional information not in the ms, or briefly explain why the item is not applicable/relevant for your study

"We started to recruit participants from June 2013...We stopped the recruitment in December 2018."

"After the baseline assessment, participants were invited to complete 11 CBM training sessions and assessments at midtraining, posttraining, and follow-ups at 1, 2, and 3 months."

14a-i) Indicate if critical “secular events” fell into the study period

Indicate if critical “secular events” fell into the study period, e.g., significant changes in Internet resources available or “changes in computer hardware or Internet delivery resources”

	1	2	3	4	5	
subitem not at all important	○	○	◉	○	○	essential

Does your paper address subitem 14a-i?

Copy and paste relevant sections from the manuscript (include quotes in quotation marks "like this" to indicate direct quotes from your manuscript), or elaborate on this item by providing additional information not in the ms, or briefly explain why the item is not applicable/relevant for your study

N/A.

14b) Why the trial ended or was stopped (early)

Does your paper address CONSORT subitem 14b? *

Copy and paste relevant sections from the manuscript (include quotes in quotation marks "like this" to indicate direct quotes from your manuscript), or elaborate on this item by providing additional information not in the ms, or briefly explain why the item is not applicable/relevant for your study

We stopped recruitment in December 2018 as we reached over 75 participants per training condition as we planed.

15) A table showing baseline demographic and clinical characteristics for each group

NPT: When applicable, a description of care providers (case volume, qualification, expertise, etc.) and centers (volume) in each group

Does your paper address CONSORT subitem 15? *

Copy and paste relevant sections from the manuscript (include quotes in quotation marks "like this" to indicate direct quotes from your manuscript), or elaborate on this item by providing additional information not in the ms, or briefly explain why the item is not applicable/relevant for your study

"Baseline characteristics of the final sample per condition can be found in Table 1."

15-i) Report demographics associated with digital divide issues

In ehealth trials it is particularly important to report demographics associated with digital divide issues, such as age, education, gender, social-economic status, computer/Internet/ehealth literacy of the participants, if known.

	1	2	3	4	5	
subitem not at all important	○	○	○	●	○	essential

Does your paper address subitem 15-i? *

Copy and paste relevant sections from the manuscript (include quotes in quotation marks "like this" to indicate direct quotes from your manuscript), or elaborate on this item by providing additional information not in the ms, or briefly explain why the item is not applicable/relevant for your study

"Baseline characteristics of the final sample per condition can be found in Table 1. Overall, participants' baseline characteristics did not differ among the training conditions, with the exception of gender (see Table 1). Adding gender as a covariate to the models did not affect relative model fit or the significance of any relevant parameters."

16) For each group, number of participants (denominator) included in each analysis and whether the analysis was by original assigned groups

16-i) Report multiple “denominators” and provide definitions

Report multiple “denominators” and provide definitions: Report N’s (and effect sizes) “across a range of study participation [and use] thresholds” [1], e.g., N exposed, N consented, N used more than x times, N used more than y weeks, N participants “used” the intervention/comparator at specific pre-defined time points of interest (in absolute and relative numbers per group). Always clearly define “use” of the intervention.

	1	2	3	4	5	
subitem not at all important	○	○	○	◉	○	essential

Does your paper address subitem 16-i? *

Copy and paste relevant sections from the manuscript (include quotes in quotation marks "like this" to indicate direct quotes from your manuscript), or elaborate on this item by providing additional information not in the ms, or briefly explain why the item is not applicable/relevant for your study

"The Consolidated Standards of Reporting Trials (CONSORT) flow diagram is reported in Figure 1. The final sample comprised 504 adult smokers who were seeking online help for quitting smoking."

"Since the enrollment occurred online, additional actions were taken to check multiple identities. Participants who used similar usernames, email addresses, and internet protocol addresses were excluded in the data analysis stage (n=8). In addition, participants who self-reported that they already quit smoking before the training were also excluded in the data analysis stage (n=25; see Figure 1)."

16-ii) Primary analysis should be intent-to-treat

Primary analysis should be intent-to-treat, secondary analyses could include comparing only “users”, with the appropriate caveats that this is no longer a randomized sample (see 18-i).

	1	2	3	4	5	
subitem not at all important	○	○	○	◉	○	essential

Does your paper address subitem 16-ii?

Copy and paste relevant sections from the manuscript (include quotes in quotation marks "like this" to indicate direct quotes from your manuscript), or elaborate on this item by providing additional information not in the ms, or briefly explain why the item is not applicable/relevant for your study

"To test the training effects, a Multilevel Modeling (MLM) approach was adopted, which allows for intention-to-treat (ITT) analysis including all available data and takes the clustering of data by participants into account [49]."

Also see Figure 1 (CONSORT flow diagram) for modified intention-to-treat analysis.

17a) For each primary and secondary outcome, results for each group, and the estimated effect size and its precision (such as 95% confidence interval)

Does your paper address CONSORT subitem 17a? *

Copy and paste relevant sections from the manuscript (include quotes in quotation marks "like this" to indicate direct quotes from your manuscript), or elaborate on this item by providing additional information not in the ms, or briefly explain why the item is not applicable/relevant for your study

"The summary statistics of all outcomes by condition, time phase, and assessment time points are reported in Table 3...The results of the MLM analyses to test the training effects (omnibus effects) on all outcomes are reported in Table 4. The full MLM models for all outcomes can be found in Multimedia Appendix 6."

17a-i) Presentation of process outcomes such as metrics of use and intensity of use

In addition to primary/secondary (clinical) outcomes, the presentation of process outcomes such as metrics of use and intensity of use (dose, exposure) and their operational definitions is critical. This does not only refer to metrics of attrition (13-b) (often a binary variable), but also to more continuous exposure metrics such as “average session length”. These must be accompanied by a technical description how a metric like a “session” is defined (e.g., timeout after idle time) [1] (report under item 6a).

	1	2	3	4	5	
subitem not at all important	○	○	○	○	◉	essential

Does your paper address subitem 17a-i?

Copy and paste relevant sections from the manuscript (include quotes in quotation marks "like this" to indicate direct quotes from your manuscript), or elaborate on this item by providing additional information not in the ms, or briefly explain why the item is not applicable/relevant for your study

"The Consolidated Standards of Reporting Trials (CONSORT) flow diagram is reported in Figure 1."

"On average, participants completed 2.75 (SD 3.45) out of the 11 training sessions, which did not differ between conditions (F3,500=0.11, P=.95). Of the final sample, 67.1% (338/504) completed at least one training session, 21.4% (108/504) completed at least five training sessions, and 10.7% (54/504) completed all 11 training sessions, all of which did not differ between conditions (X23=2.5, P=.48; X23=0.9, P=.83; X23=1.5, P=.68). On average, the training interval was 5.57 days (SD 4.64), which did not differ between conditions (F3,332=1.32, P =.27)."

17b) For binary outcomes, presentation of both absolute and relative effect sizes is recommended

Does your paper address CONSORT subitem 17b? *

Copy and paste relevant sections from the manuscript (include quotes in quotation marks "like this" to indicate direct quotes from your manuscript), or elaborate on this item by providing additional information not in the ms, or briefly explain why the item is not applicable/relevant for your study

"The summary statistics of all outcomes by condition, time phase, and assessment time points are reported in Table 3...The results of the MLM analyses to test the training effects (omnibus effects) on all outcomes are reported in Table 4. The full MLM models for all outcomes can be found in Multimedia Appendix 6."

18) Results of any other analyses performed, including subgroup analyses and adjusted analyses, distinguishing pre-specified from exploratory

Does your paper address CONSORT subitem 18? *

Copy and paste relevant sections from the manuscript (include quotes in quotation marks "like this" to indicate direct quotes from your manuscript), or elaborate on this item by providing additional information not in the ms, or briefly explain why the item is not applicable/relevant for your study

"Summary of Additional Analyses
The methods and results for testing training effects on the additional secondary outcomes (ie, craving, depression severity, and motivation to quit smoking) and for the exploratory moderation analysis on participants' awareness of CBM condition are reported in the Multimedia Appendices 1 and 2. The main findings were..."

18-i) Subgroup analysis of comparing only users

A subgroup analysis of comparing only users is not uncommon in ehealth trials, but if done, it must be stressed that this is a self-selected sample and no longer an unbiased sample from a randomized trial (see 16-iii).

	1	2	3	4	5	
subitem not at all important	○	○	◉	○	○	essential

Does your paper address subitem 18-i?

Copy and paste relevant sections from the manuscript (include quotes in quotation marks "like this" to indicate direct quotes from your manuscript), or elaborate on this item by providing additional information not in the ms, or briefly explain why the item is not applicable/relevant for your study

Your answer

19) All important harms or unintended effects in each group

(for specific guidance see CONSORT for harms)

Does your paper address CONSORT subitem 19? *

Copy and paste relevant sections from the manuscript (include quotes in quotation marks "like this" to indicate direct quotes from your manuscript), or elaborate on this item by providing additional information not in the ms, or briefly explain why the item is not applicable/relevant for your study

Participants did not report any important harms or unintended effects of the training.

19-i) Include privacy breaches, technical problems

Include privacy breaches, technical problems. This does not only include physical “harm” to participants, but also incidents such as perceived or real privacy breaches [1], technical problems, and other unexpected/unintended incidents. “Unintended effects” also includes unintended positive effects [2].

	1	2	3	4	5	
subitem not at all important	○	○	●	○	○	essential

Does your paper address subitem 19-i?

Copy and paste relevant sections from the manuscript (include quotes in quotation marks "like this" to indicate direct quotes from your manuscript), or elaborate on this item by providing additional information not in the ms, or briefly explain why the item is not applicable/relevant for your study

There were no privacy breaches and any technical problems related to privacy breaches.

19-ii) Include qualitative feedback from participants or observations from staff/researchers

Include qualitative feedback from participants or observations from staff/researchers, if available, on strengths and shortcomings of the application, especially if they point to unintended/unexpected effects or uses. This includes (if available) reasons for why people did or did not use the application as intended by the developers.

	1	2	3	4	5	
subitem not at all important	○	○	○	●	○	essential

Does your paper address subitem 19-ii?

Copy and paste relevant sections from the manuscript (include quotes in quotation marks "like this" to indicate direct quotes from your manuscript), or elaborate on this item by providing additional information not in the ms, or briefly explain why the item is not applicable/relevant for your study

"Training Evaluation and Quitting the Intervention
In total, 19.6% (99/504) of the final sample provided the training evaluation...Since there were no differences in TEQ responses between training versions for both AtBM and ApBM (results are reported in Multimedia Appendix 5), the overall responses to the TEQ are summarized in Table 2. "

DISCUSSION

22) Interpretation consistent with results, balancing benefits and harms, and considering other relevant evidence

NPT: In addition, take into account the choice of the comparator, lack of or partial blinding, and unequal expertise of care providers or centers in each group

22-i) Restate study questions and summarize the answers suggested by the data, starting with primary outcomes and process outcomes (use)

Restate study questions and summarize the answers suggested by the data, starting with primary outcomes and process outcomes (use).

	1	2	3	4	5	
subitem not at all important	○	○	○	◉	○	essential

Does your paper address subitem 22-i? *

Copy and paste relevant sections from the manuscript (include quotes in quotation marks "like this" to indicate direct quotes from your manuscript), or elaborate on this item by providing additional information not in the ms, or briefly explain why the item is not applicable/relevant for your study

"This double-blind RCT tested the individual and combined effects of Web-based AtBM and ApBM in adult smokers seeking online help for quitting smoking. Against our expectations, we did not find evidence for the effectiveness of neither CBM trainings nor their combination, compared with their respective sham version, in improving any of the smoking-related outcomes. In addition, neither did any of the CBM training conditions change the targeted cognitive biases. Compliance to the intervention was very low as only 10.7% (54/504) of participants completed all training sessions, and 8.3% (42/504) of the participants completed the follow-up assessments, suggesting that the Web-based intervention was not well accepted."

22-ii) Highlight unanswered new questions, suggest future research

Highlight unanswered new questions, suggest future research.

	1	2	3	4	5	
subitem not at all important	○	○	○	●	○	essential

Does your paper address subitem 22-ii?

Copy and paste relevant sections from the manuscript (include quotes in quotation marks "like this" to indicate direct quotes from your manuscript), or elaborate on this item by providing additional information not in the ms, or briefly explain why the item is not applicable/relevant for your study

For example:
"Thus, future online CBM studies may benefit by incorporating online CBM training with online standard treatment and additional remote support from therapists as a treatment package."

"To specifically evaluate clinical effects of CBM, one solution could be to carefully choose a more appropriate control condition, and another solution could be to make the active training more meaningful to the participants; for example, by training participants to approach (personalized) meaningful stimuli rather than neutral stimuli in addition to training them to avoid smoking stimuli [63], or by providing positive or relevant rewards to reinforce newly learned behaviors (eg, avoid smoking stimuli) during training [64]."

"In addition, to obtain a more representative sample, especially those who left the intervention without providing an evaluation of the intervention should be approached. Future research could adopt the strategy of monetary incentives to increase these response rates [69] or make more effort to interview the dropouts to understand their needs and thoughts to improve the intervention."

20) Trial limitations, addressing sources of potential bias, imprecision, and, if relevant, multiplicity of analyses

20-i) Typical limitations in ehealth trials

Typical limitations in ehealth trials: Participants in ehealth trials are rarely blinded. Ehealth trials often look at a multiplicity of outcomes, increasing risk for a Type I error. Discuss biases due to non-use of the intervention/usability issues, biases through informed consent procedures, unexpected events.

	1	2	3	4	5	
subitem not at all important	○	○	○	○	◉	essential

Does your paper address subitem 20-i? *

Copy and paste relevant sections from the manuscript (include quotes in quotation marks "like this" to indicate direct quotes from your manuscript), or elaborate on this item by providing additional information not in the ms, or briefly explain why the item is not applicable/relevant for your study

See the section "Limitations and Future Research"

21) Generalisability (external validity, applicability) of the trial findings

NPT: External validity of the trial findings according to the intervention, comparators, patients, and care providers or centers involved in the trial

21-i) Generalizability to other populations

Generalizability to other populations: In particular, discuss generalizability to a general Internet population, outside of a RCT setting, and general patient population, including applicability of the study results for other organizations

	1	2	3	4	5	
subitem not at all important	○	○	○	◉	○	essential

Does your paper address subitem 21-i?

Copy and paste relevant sections from the manuscript (include quotes in quotation marks "like this" to indicate direct quotes from your manuscript), or elaborate on this item by providing additional information not in the ms, or briefly explain why the item is not applicable/relevant for your study

"The most notable limitation of this study is the low training adherence and the high dropout rates. Although high dropout rates are similar to the few published Web-based CBM studies [54,65] and are very common in online interventions [60,66,67], this issue may have limited the validity of the results and caused power issues in this study."

21-ii) Discuss if there were elements in the RCT that would be different in a routine application setting

Discuss if there were elements in the RCT that would be different in a routine application setting (e.g., prompts/reminders, more human involvement, training sessions or other co-interventions) and what impact the omission of these elements could have on use, adoption, or outcomes if the intervention is applied outside of a RCT setting.

	1	2	3	4	5	
subitem not at all important	○	○	○	○	◉	essential

Does your paper address subitem 21-ii?

Copy and paste relevant sections from the manuscript (include quotes in quotation marks "like this" to indicate direct quotes from your manuscript), or elaborate on this item by providing additional information not in the ms, or briefly explain why the item is not applicable/relevant for your study

"A major difference is that CBM is normally administrated as an add-on intervention to the standard treatment in the clinical setting (eg, CBT [17,18,28,29]), whereas as a stand-alone or primary intervention in the online setting (as in this study)...A related difference is that, in a clinical setting, CBM is administrated in a guided environment (ie, with the support of the therapists), while an unguided environment online fully relies on participants’ autonomy, self-reliance, and self-discipline [57]...In addition, compared with clinical settings, participants recruited online are more heterogeneous in terms of severity of symptoms..."

OTHER INFORMATION

23) Registration number and name of trial registry

Does your paper address CONSORT subitem 23? *

Copy and paste relevant sections from the manuscript (include quotes in quotation marks "like this" to indicate direct quotes from your manuscript), or elaborate on this item by providing additional information not in the ms, or briefly explain why the item is not applicable/relevant for your study

Netherlands Trial Register (NTR4678)

24) Where the full trial protocol can be accessed, if available

Does your paper address CONSORT subitem 24? *

Cite a Multimedia Appendix, other reference, or copy and paste relevant sections from the manuscript (include quotes in quotation marks "like this" to indicate direct quotes from your manuscript), or elaborate on this item by providing additional information not in the ms, or briefly explain why the item is not applicable/relevant for your study

N/A.

25) Sources of funding and other support (such as supply of drugs), role of funders

Does your paper address CONSORT subitem 25? *

Copy and paste relevant sections from the manuscript (include quotes in quotation marks "like this" to indicate direct quotes from your manuscript), or elaborate on this item by providing additional information not in the ms, or briefly explain why the item is not applicable/relevant for your study

"Acknowledgments:
SW was supported by China Scholarship Council (CSC). HL was supported by Research Priority Area Yield, University of Amsterdam. RW was supported by a VICI grant (453-08-001) from the Dutch National Science Foundation, N.W.O."

X27) Conflicts of Interest (not a CONSORT item)

X27-i) State the relation of the study team towards the system being evaluated

In addition to the usual declaration of interests (financial or otherwise), also state the relation of the study team towards the system being evaluated, i.e., state if the authors/evaluators are distinct from or identical with the developers/sponsors of the intervention.

	1	2	3	4	5	
subitem not at all important	○	○	○	◉	○	essential

Does your paper address subitem X27-i?

Copy and paste relevant sections from the manuscript (include quotes in quotation marks "like this" to indicate direct quotes from your manuscript), or elaborate on this item by providing additional information not in the ms, or briefly explain why the item is not applicable/relevant for your study

"Conflicts of Interests
None declared."

About the CONSORT EHEALTH checklist

As a result of using this checklist, did you make changes in your manuscript? *

- ◉ yes, major changes
- ○ yes, minor changes
- ○ no

What were the most important changes you made as a result of using this checklist?

Format

How much time did you spend on going through the checklist INCLUDING making changes in your manuscript *

One week

As a result of using this checklist, do you think your manuscript has improved? *

- (●) yes
- ( ) no
- ( ) Other:

Would you like to become involved in the CONSORT EHEALTH group?

This would involve for example becoming involved in participating in a workshop and writing an "Explanation and Elaboration" document

- ( ) yes
- (●) no
- ( ) Other:

Any other comments or questions on CONSORT EHEALTH

Your answer

STOP - Save this form as PDF before you click submit

To generate a record that you filled in this form, we recommend to generate a PDF of this page (on a Mac, simply select "print" and then select "print as PDF") before you submit it.

When you submit your (revised) paper to JMIR, please upload the PDF as supplementary file.

Don't worry if some text in the textboxes is cut off, as we still have the complete information in our database. Thank you!

Final step: Click submit !

Click submit so we have your answers in our database!

Submit

Never submit passwords through Google Forms.

This content is neither created nor endorsed by Google. Report Abuse - Terms of Service - Privacy Policy

Google Forms
